# Porous Transport Layers for Anion Exchange Membrane Water Electrolysis: The Impact of Morphology and Composition

**DOI:** 10.1021/acselectrochem.4c00207

**Published:** 2025-02-07

**Authors:** Melissa E. Kreider, Ambar R. Maldonado Santos, Arielle L. Clauser, Matthew E. Sweers, Leiming Hu, Emily K. Volk, Ai-Lin Chan, Joshua D. Sugar, Shaun M. Alia

**Affiliations:** † Chemistry and Nanoscience Center, 53405 National Renewable Energy Laboratory, Golden, Colorado 80401, United States; ‡ Department of Chemistry, University of Puerto Rico - Rio Piedras, San Juan, Puerto Rico 00931, United States; § 1105 Sandia National Laboratories, Livermore, California 94551, United States; ∥ Chemical Sciences and Engineering Division, 1291 Argonne National Laboratory, Lemont, Illinois 60439, United States; ⊥ Advanced Energy Systems Graduate Program, 3557 Colorado School of Mines, Golden, Colorado 80401, United States

**Keywords:** hydrogen, water electrolysis, anion exchange membrane water electrolyzer, porous transport layer, catalyst layer, corrosion

## Abstract

Anion exchange membrane water electrolysis (AEMWE) is an emerging technology for the low-cost production of hydrogen. However, the efficiency and durability of AEMWE devices is currently insufficient to compete with other low-temperature electrolysis technologies. The porous transport layer (PTL) is a critical cell component that remains relatively unoptimized for AEMWE. In this study, we demonstrate that device performance is significantly affected by the morphology and composition of the PTL. For Ni fiber-based PTLs with a ∼2 μm Co_3_O_4_ oxygen evolution reaction catalyst layer, decreasing the pore size and porosity resulted in a 20% increase in current density at 2 V in 1 M KOH supporting electrolyte. Alloy PTLs with even lower porosity had a higher performance; in particular, the stainless steel PTL gave an 80% increase in current density relative to Ni. Without Co_3_O_4_, the alloy PTLs still demonstrated high activity, indicating that the PTL material was catalytically active. However, characterization of the electrode and electrolyte after testing indicated that the alloy PTLs also underwent restructuring and corrosion processes that may limit long-term stability. This study demonstrates that the design of PTLs with improved morphology and composition is an important area of focus to achieve AEMWE performance targets.

## Graphical abstract

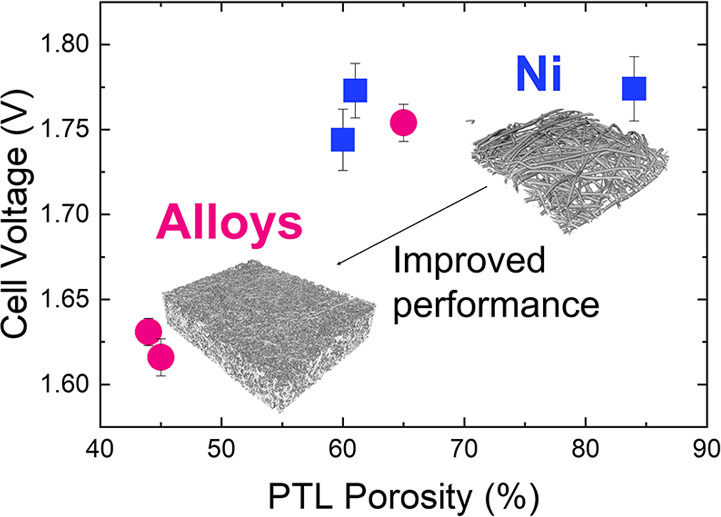

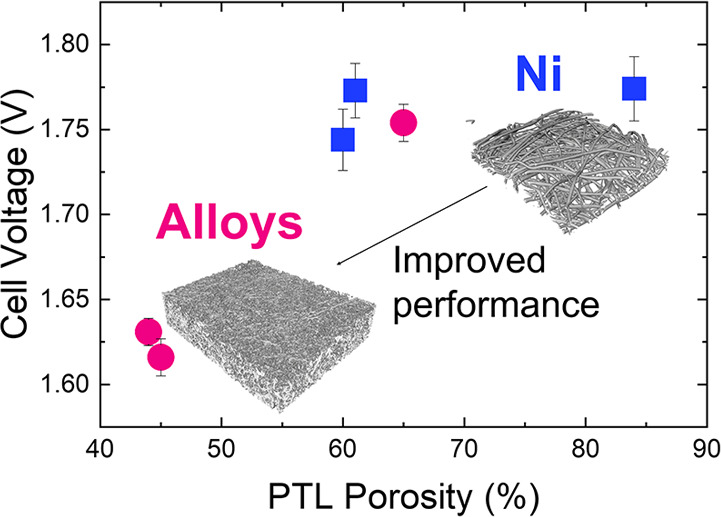

## Introduction

Hydrogen is poised to play an important role in the future energy system as a potential low cost energy carrier and feedstock for the transportation, chemical, and energy storage sectors.^1^ While hydrogen production methods via the reforming of natural gas are inexpensive, low cost electricity inputs can enable water electrolyzers (WE) to be competitive, provided that capital cost reductions can be achieved.^1^ Liquid alkaline (LA) and proton exchange membrane (PEM) electrolyzers have been commercialized as hydrogen production technologies, however they have limitations in terms of efficiency and the use of expensive, platinum group metal (PGM) components, respectively.^2,3^ Anion exchange membrane water electrolyzers (AEMWE) have gained significant interest due to the alkaline environment that enables the use of earth abundant, PGM-free catalysts and other stack materials, potentially lowering the capital cost, while the use of a solid polymer membrane electrolyte in a zero-gap architecture allows for higher current densities as well as lower hydrogen crossover rates compared to traditional LA.^4–10^ Despite these potential advantages, AEMWE technology is currently in an early stage of development, with improvements in efficiency and durability needed to be competitive. Specifically, the US Department of Energy has released guidelines for AEMWE performance, targeting 2 A/cm^2^ at 1.8 V with less than 4 μV/h degradation rate.^11^ Several devices have been reported in the literature that meet this initial performance target, but very few even approach the degradation rate requirement at industrially relevant current densities^12,13^

In an AEMWE, the membrane electrode assembly (MEA) consists of the membrane, catalyst layers, and porous transport layers (PTLs). In order for the hydrogen evolution reaction (HER) and oxygen evolution reaction (OER) to produce hydrogen and oxygen at the cathode and anode, respectively, electrons, hydroxide ions, water, and gaseous products must be transported to and from the catalysts’ active sites. The electrode structure, including the PTL, ionomer type and loading, and catalyst morphology, will affect the accessibility of catalyst sites.^14^ These considerations are particularly important for the anode because device performance is typically limited by the sluggish OER kinetics, with additional disadvantages of lower electronic conductivity and surface area for the oxide catalysts, which both limit the number of accessible active sites.^15,16^ Therefore, improvements in the anode PTL that improve catalyst utilization can yield large benefits in device efficiency.^17^ The PTL has five important functions: transporting (1) electrons, (2) KOH/water, and (3) O_2_ to and away from catalyst active sites; (4) assisting in thermal management through heat transfer away from the catalyst; and (5) providing mechanical strength to the membrane.^18,19^ PTLs must be optimized to serve all of these roles simultaneously, and it is therefore critical to understand which PTL properties limit performance.

The main PTL design challenge has been identified as providing sufficient electronic conductivity to the catalyst layer while also efficiently removing O_2_ bubbles.^20^ Several studies have shown that in-plane electronic conductivity through the catalyst layer is very inefficient, such that catalyst sites that are not directly in contact with the PTL may be electronically isolated and inactive.^17–19,21^ This is particularly challenging for low-loading, nonuniform catalyst layers.^22–26^ To minimize the inactive catalyst area underneath the pores of the PTL, it is desirable to maximize interfacial contact area with the catalyst layer by using PTLs with small pore sizes and low porosity.^19,24,27^ On the other hand, PTLs with larger pores and higher porosity have been found to improve mass transport of gas bubbles, which is also critical to prevent blocking of active sites and membrane dry out.^17,28^ Hydrophilic and low tortuosity PTLs have also been found to improve mass transport by decreasing the bubble detachment time and the resistance to flow, respectively.^29–32^ Bilayer structured materials, such as those made with the addition of a microporous layer, have shown promise for balancing the needs for large interfacial contact area and efficient bubble transport.^28,32–34^ The morphology of the PTL, particularly the pore size, porosity, and tortuosity, is thus critical to electrolyzer performance.

PTLs for AEMWE present a few additional challenges. First, most of the commercially available AEMWE PTLs have been adapted from other applications, such as filtration media, and therefore often have sub-optimal morphological properties. Second, due to the thinness and limited mechanical stability of current anion exchange membranes, it is common to use a catalyst-coated substrate (CCS) MEA architecture. In this configuration, the PTL morphology will directly determine the catalyst layer morphology, with large gaps possible if the PTL pores are significantly larger than the catalyst layer thickness.^30^ Next, the typical Ni-based PTL materials are prone to oxidation under reaction conditions, and without a PGM coating this can lead to increases in ohmic resistance over time.^23,35^ Finally, there are trade-offs between performance and durability that must be considered. There is interest in using metal alloy PTLs, such as stainless steel, which can be cheaper than the standard Ni materials and are already available with decreased fiber and pore dimensions for improved interfacial contact with the catalyst layer.^13^ Just as transition metal dopants in Ni oxide catalysts improve OER activity, PTLs composed of a mixture of Cr, Fe, and Ni have shown improved performance compared to Ni alone.^33,36^ Despite this advantage, the alloy PTLs may also suffer from increased corrosion over time. In PEMWE, migration of impurities such as Fe to the membrane and cathode and mechanical restructuring that leads to loss of contact with the catalyst layer have been identified as important degradation mechanisms.^37–41^ To direct future PTL development efforts, better understanding of the relative importance of morphology and composition for initial performance is needed, as is an assessment of PTL degradation mechanisms in AEMWE.

In this work, we investigate the impact of the anode PTL on AEMWE performance in supporting electrolyte, with and without a Co_3_O_4_ catalyst layer. Different aspects of the PTL are studied using six commercially available PTLs with varying morphology (e.g., thickness, porosity, pore size) and composition (e.g., Ni, alloys). Tomography, microscopy, and voltage loss breakdown analysis (VBA) are used to relate the material properties of the PTLs to their impact on AEMWE performance. It is found that decreased fiber and pore sizes improve the homogeneity of the Co_3_O_4_ catalyst layer and decrease the catalyst layer resistance and mass transport losses. Compared to the standard Ni PTL, the alloy PTLs exhibit significantly improved kinetics and mass transport. To distinguish between the impact of the PTL on the catalyst layer and the activity of the PTL itself, the PTLs are also studied without an additional catalyst layer. Cyclic voltammograms (CVs) show that the PTL impacts redox features and capacitance more than the presence or absence of Co_3_O_4_, indicating that much of the electrochemically accessible surface area is from the PTL. Similar activity trends are observed, with stainless steel reaching an extremely high performance of 3.8 A/cm^2^ at 2 V without a catalyst layer. While the alloy PTLs show only modest voltage increase rates of ∼1 mV/h over 100 h at 1 A/cm^2^, significant PTL degradation including segregation, corrosion, and restructuring is observed. We discuss trade-offs between activity and durability for the alloy PTLs, including the impact of dissolved metal species and loss of mechanical integrity, for long-term operation. These findings suggest PTL-only anodes as a potential route to avoid issues of ionomer and catalyst-layer instability, as well as costs associated with catalyst layer processing. The rational design and development of improved PTL materials for AEMWE is a promising route to achieve efficiency and durability targets.

## Methods

### Catalyst Materials

Commercial Co_3_O_4_ (US Research Nanomaterials Inc., 99%) and Pt/C (47% Pt, TKK TEC10E50E) catalysts were used without further treatment. Fiber PTLs (Bekaert) are Currento 2Ni 18-0.2 (Ni 200), Currento 2Ni 18-0.25 (Ni 250), Currento 2Ni 18-0.5 (Ni 500), Bekipor ST10AL3 Alloy HR (HR), Bekipor XL601S AISI 316L (SS), and Bekipor ST30AL3 Inconel 601 (Inconel). The composition and morphology of the PTLs are summarized in Table 1.

**Table 1:** Summary of PTL Material Properties

PTL	composition	thickness (μm)	fiber dimension (μm)	porosity (%)	avg pore size (μm)
Ni 200	100% Ni	215	20	84	44
Ni 250	100% Ni	270	20	61	18
Ni 500	100% Ni	530	20	60	16
stainless steel (SS)	65% Fe, 18% Cr, 14% Ni, 3% Mo	430	2 (top layer)	44	9
high resistance alloy (HR)	60% Ni, 22% Cr, 16% Mo, 2% Fe	290	4	45	7
inconel (Inc)	60% Ni, 25% Cr, 15% Fe	600	12	65	22

### Physical and Chemical Characterization

X-ray diffraction (XRD) patterns were collected using a Bruker D8 Discover with Cu K-α radiation (λ = 0.15406 nm) in the 2θ range between 13.5 and 88°. Reported PTL composition and catalyst loadings on the anode and cathode were taken as the average of 3 measurements with 30 s exposure using X-ray fluorescence (Fischer XDV-SDD XRF). Micro X-ray computed tomography (micro-XCT) was utilized to examine the porosity and pore size distribution of the anode PTL. The characterizations were carried out using a NSI X3000 instrument (North Star Imaging, USA). Each PTL was sectioned into a sample area of 1.5 x 2.0 mm and mounted on a rotating stage. The scan parameters included a source voltage of 80 keV and a target current of 40 μA, with a field of view of 1.5 x 1.5 mm and an effective pixel size of 1 μm. Scans were performed with 1081 projections over a 360° rotation, and each projection was averaged 100 times with a 0.5 second exposure per image. A filter back projection (FBP) algorithm was employed for image reconstruction. To ensure consistency in reconstructed voxel size, the source and detector positions remained fixed for all GDE scans. The reconstructed 3D volumes were then segmented and analyzed using custom-developed MATLAB code. A Keyence VHX 7000 digital microscope was used to produce optical images of the PTLs. Top-down and cross-section scanning electron microscopy (SEM) images were collected using an FEI Helios NanoLab 660 DualBeam SEM/focused ion beam (FIB) instrument with a solid-state circular backscatter detector operated at 5 kV and 0.2 nA and an Everhart-Thornley detector for secondary electron images. Cross-section samples were prepared using the FIB. Energy dispersive X-ray spectroscopy (EDS) element maps were collected using an Oxford 170 mm^2^ Ultim Max SDD EDS detector at 15 kV and 6.4 nA with Oxford’s AZtec software. Soft X-ray absorption spectroscopy (XAS) measurements were conducted at the Stanford Synchrotron Radiation Light Source (SSRL) on BL 8-2 in total electron and fluorescence yield modes. The Cr, Fe, Co, and Ni L-edge spectra were analyzed using the Athena software package,^42^ with some spectra additionally processed using an IGOR Pro macro to normalize the integrated area. A Thermo Scientific iCAP Q instrument was used for inductively coupled plasma-mass spectrometry (ICP-MS) in kinetic energy discrimination mode using He cell gas. Aliquots were taken from the three-electrode cell and single-cell electrolyte reservoir and diluted 100× with 2% HNO_3_ (Fischer Chemical, Optima Grade, 67–70%) prior to analysis. Standards of Cr, Fe, Co, Ni, Mo, and Pt from 1 to 25 ppb in 2% HNO_3_ were used for calibration, while diluted 1 M KOH was used as an additional blank to account for impurities in the electrolyte.

### Electrochemical Characterization

Half-cell measurements of the PTLs were conducted in a glass rotating disk electrode (RDE) cell with a Au counter electrode and reversible hydrogen electrode (RHE) reference. The 1 M KOH (EMD Millipore, Emsure grade) electrolyte was bubbled with N_2_ for 10 min to removed dissolved gases. The PTLs were placed in the electrolyte, without rotation, with an active area of ∼0.8 cm^2^. The OER activity and capacitance were assessed using linear sweep voltammetry (LSV) and CV. An Autolab PGSTAT302N potentiostat (Eco Chemie, Metrohm Autolab) was used for all half-cell measurements.

For single-cell measurements, the 5 cm^2^ active area MEAs consisted of Pt/C cathode, PiperION-A TP-85 membrane (80 μm) and ionomer (Versogen), and various anode PTLs with or without a Co_3_O_4_ catalyst layer. The Pt/C catalyst layer was sprayed onto a carbon paper gas diffusion layer (Avcarb MGL280, 80280-40) using an airbrush, targeting a loading of 0.3 mg_Pt_/cm^2^ and 30 wt % ionomer-to-(ionomer + catalyst) ratio, as described previously.^22^ The Co_3_O_4_ catalyst layer was sprayed onto the PTL by the same method, with a target loading of 0.5 mg_Co_/cm^2^ and 30 wt % ionomer. For the tests without an anode catalyst layer, the PTLs were used without modification. Before assembly, the membranes were ion exchanged from carbonate to hydroxide form in 3 M KOH for 48 h and the electrodes containing ionomer were ion exchanged in 0.5 M KOH for 10 min.

The AEMWE tests were conducted in custom-built, 25 cm^2^ hardware with stainless steel (316L) end plates, Au-coated current collectors, and triple-serpentine Ni flow fields. Approximately 20% compression was achieved using PTFE gaskets (thickness adjusted to account for varying PTL thickness) and 4.5 N·m torque. 1 M KOH electrolyte was supplied to the anode and cathode at 50 mL/min each, and all tests were conducted at a cell temperature of 80 °C. An Autolab PGSTAT302N potentiostat with 20 A booster (Eco Chemie, Metrohm Autolab) was used for all AEMWE measurements. The gaskets were used to restrict the active area to 5 cm^2^ to allow for measurements up to 4 A/cm^2^ current density with the 20 A limit. For the tests with a Co_3_O_4_ anode catalyst layer, the MEA was conditioned at 2 V for 2 h, while no conditioning step was employed for the PTL-only tests. Potentiostatic polarization curves were taken via 2 min holds at voltages between 1.4 and 2 V. The thermodynamic water splitting potential under operating conditions was calculated to be 1.178 V, as reported previously.^22,43,44^ Electrochemical impedance spectroscopy (EIS) was performed at the same voltage steps used in the polarization curve at frequencies from 18 to 1 kHz with an AC amplitude of 10 mV, with additional measurements taken at non-Faradaic voltages of 1.25, 1.3, and 1.35 V to determine the catalyst layer resistance (CLR) due to in-plane electronic and through-plane ionic and electronic resistances.^45,46^ Briefly, the non-Faradaic impedance is modeled with a transmission line, using an open source impedance fitting (OSIF) tool to determine the CLR.^47^ The CLR is associated with voltage losses due to lower catalyst utilization. The high frequency resistance (HFR) was determined at every voltage via interpolation of the high frequency region in the EIS spectra, as well as using the OSIF tool to model it. Tafel slopes and intercepts, or exchange current densities, were calculated using the HFR-free voltage in the current range of 5 to 50 mA/cm^2^. It should be noted that this is an “effective” Tafel slope, encapsulating effects from the anode and the cathode catalysts, as well as transport layers, and thus should not be considered to provide mechanistic insight. Finally, CVs were measured from 0.025 to 1.4 V at 20, 50, and 100 mV/s.

## Results and Discussion

### PTL Characterization

The anode PTL serves important functions for the transport of electrons, electrolyte, and gaseous products that strongly impact AEMWE performance. In supporting electrolyte, the PTL can also provide active sites and contribute directly to the OER activity. In this study, six PTLs of varying thickness, pore size, porosity, and composition are studied for their impact on AEMWE performance, with and without catalyst layers. Three of the PTLs are Ni fibers, with nominal thickness ranging from 200 to 500 μm and fiber dimensions of 20 μm. As shown in Figure 1a, the X-ray diffraction (XRD) patterns for these three PTLs match well to fcc Ni (ICSD 37502), with no other phases present. Micro X-ray computed tomography (micro-XCT) shows that the ∼270 and 530 μm thick Ni PTLs (denoted Ni 250 and Ni 500, respectively) have similar porosity at ∼60% with an average pore size at 17 μm (Figure 1b,c). In contrast, the 200 μm thick Ni PTL (Ni 200) has a significantly higher porosity at 84% and larger average pore size of 44 μm.

**Figure 1:**
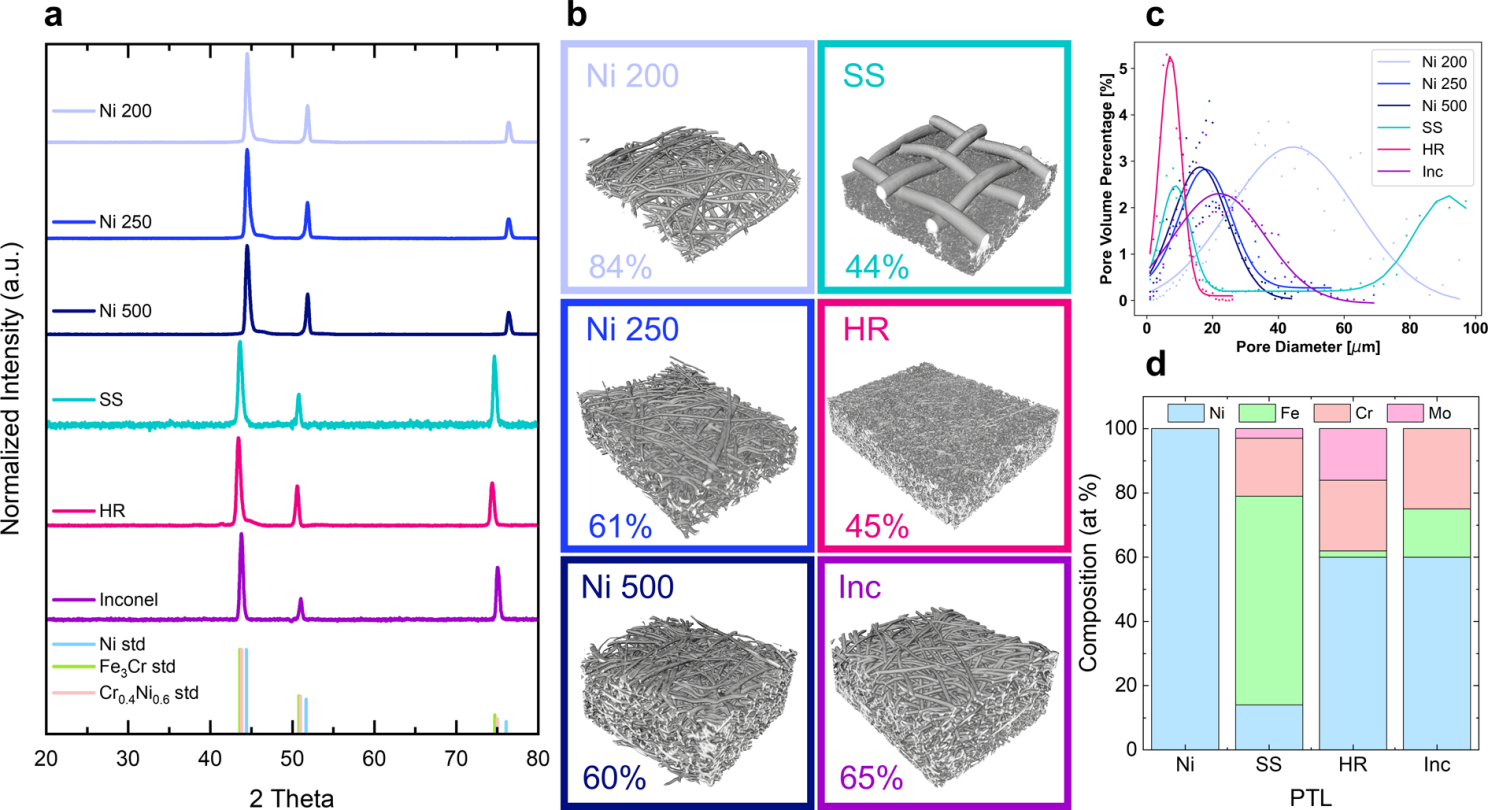
Characterization of the material properties of the bare PTLs. (a) XRD patterns for the Ni 200 (light blue), Ni 250 (blue), Ni 500 (dark blue), SS (teal), HR (pink), and Inc (purple) PTLs with reference stick patterns for Ni (sky blue, ICSD 37502), Fe_3_Cr (lime green, ICSD 253959), and Cr_0.4_Ni_0.6_ (coral, ICSD 102821). (b) Micro-XCT reconstructions with measured porosity percentage and (c) calculated pore diameters. (d) Metal composition of the Ni, SS, HR, and Inc PTLs, as measured by XRF.

The other three PTLs are composed of alloy fibers. The XRD patterns of these alloy PTLs indicate they all have a single alloy structure, no additional phases, and an fcc crystal structure (Figure 1a); SS and HR match well to a Fe_3_Cr standard (ICSD 253959), while Inconel matches the Cr_0.4_Ni_0.6_ standard (ICSD 102821). The XRF data (Figure 1d) shows that the stainless steel (SS) is 65% Fe with ∼15% Cr and Ni, while the high resistance alloy (HR) and Inconel (Inc) are composed of 60% Ni with smaller amounts of Cr, Mo, and Fe. Soft XAS at the Cr, Fe, and Ni L_III–II_ edges, measured in total electron yield (TEY) mode for surface-specific (∼top few nm) analysis,^48^ suggests that Cr and Fe exist in oxidized states at the surface of the SS and HR PTLs,^49,50^ whereas Ni appears less oxidized than a Ni­(II)O standard in all of the PTLs (Figure S1). Additionally, a comparison of the background-subtracted peak intensities of the TEY and total fluorescence yield (TFY, ∼100s of nm) signal at these edges indicates that the surfaces of the SS and HR PTLs are relatively Cr-rich (Figure S2). Compared to the Ni PTLs, the alloy PTLs are composed of smaller fibers of 12, 4, and 2 μm for Inc, HR, and SS, respectively. For Inc, this results in porosity (65%) and pore size (22 μm) that are comparable to that of Ni 250 and 500. For SS and HR, however, the porosity decreases to ∼45% with average pore size of 9 and 7 μm, respectively. It should be noted that the SS PTL includes a second layer with much larger fiber dimension and pore size (90 μm), which provides mechanical support to the finer top layer (Figure 1b,c). These properties are summarized in Table 1.

### PTL Performance with Catalyst Layer

The effect of these varied PTL properties on AEMWE performance is first studied by comparing anodes composed of Co_3_O_4_ catalyst layers (∼0.5 mg_Co_/cm^2^ loading) supported on the six PTLs. Top-down optical and SEM images clearly show the differences in fiber size, pore size, and porosity for each PTL, as well as the effects of these properties on the morphology of the catalyst layer (Figure S3). For example, the catalyst layer on the Inc PTL only has approximately 40% areal coverage, while SS has close to 90% coverage, corresponding to their differences in porosity (65% for Inc, 44% for SS). When the catalyst layer thickness is smaller than the PTL pore size, as it is for the Inc PTL with catalyst layer thickness of ∼4 μm and average pore size of 22 μm, the catalyst layer will likely not be able to cover the pore area, meaning that the PTL porosity will directly determine the catalyst area.^22^ Therefore, we expect the morphology of the PTL to have significant effects on catalyst utilization and performance. Figure 2 shows the polarization curves and EIS at 1.7 V for each Co_3_O_4_/PTL anode. For the Ni PTLs, performance varies slightly based on thickness and porosity changes. The current density at 2 V (J@2) is highest for Ni 500 at 2.0 A/cm^2^, representing increases of ∼10 and 20% over Ni 250 and Ni 200, respectively (Figure 2a). The HFR-free voltage at 1 A/cm^2^ (V@1) also decreases by 30 mV from 1.77 V for Ni 200 and Ni 250 to 1.74 V for Ni 500. These performance metrics are summarized in Table 2. The EIS results in Figure 2b show no significant differences in HFR, indicating that contact resistance has minimal effect on HFR, but the charge transfer resistance (*R*_CT_) decreases with increasing PTL thickness. Comparing the Ni 200 and Ni 500 PTLs, the combination of increased thickness, decreased porosity, and decreased pore size has a slightly positive effect on overall performance.

**Figure 2:**
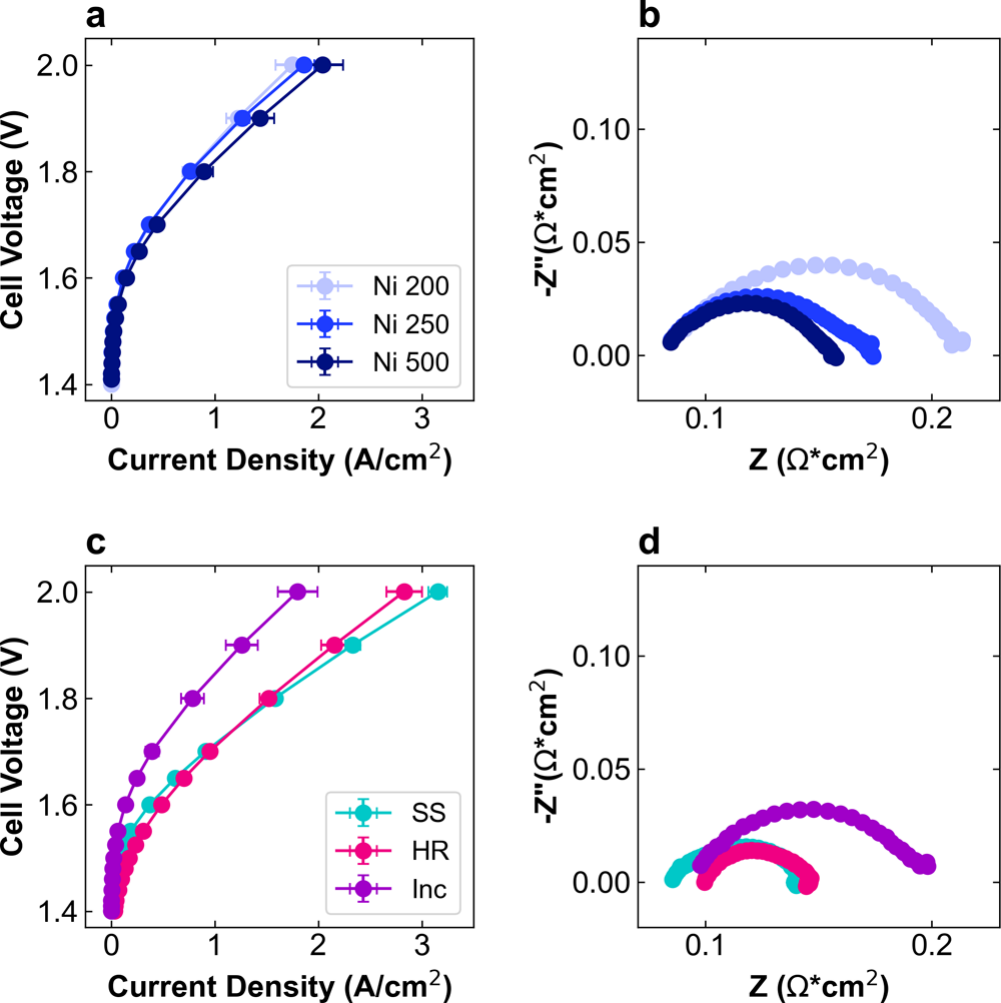
AEMWE performance of various PTLs with Co_3_O_4_ catalyst layer. (a, c) Polarization curves and (b, d) Nyquist EIS plot at 1.9 V for (a, b) Ni 200 (light blue), Ni 250 (blue), and Ni 500 (dark blue) PTLs and for (c, d) SS (teal), HR (pink), and Inc (purple) PTLs. Polarization curves are reported in triplicate from separate cell tests; EIS are from one test chosen as the representative of the average.

**Table 2:** Summary of AEMWE Performance for PTLs with a Co_3_O_4_ Catalyst Layer

PTL	J@2 (A/cm^2^)	V@1 (V HFR-free)	HFR (Ω·cm^2^)	*R*_CL_ (Ω·cm^2^)	Tafel slope (mV/dec)	*J*_o_ (μA/cm^2^)
Ni 200	1.72 ± 0.17	1.77 ± 0.02	0.084 ± 0.004	0.62 ± 0.21	95 ± 3	9.1 ± 3.9
Ni 250	1.86 ± 0.10	1.77 ± 0.02	0.074 ± 0.008	0.58 ± 0.13	102 ± 4	9.4 ± 3.0
Ni 500	2.04 ± 0.20	1.74 ± 0.02	0.077 ± 0.003	0.51 ± 0.10	89 ± 4	6.4 ± 2.3
SS	3.15 ± 0.09	1.63 ± 0.01	0.082 ± 0.005	0.28 ± 0.02	76 ± 1	3.7 ± 1.3
HR	2.83 ± 0.17	1.62 ± 0.01	0.092 ± 0.006	0.30 ± 0.09	94 ± 1	120 ± 27
Inc	1.80 ± 0.25	1.75 ± 0.01	0.092 ± 0.011	0.78 ± 0.13	93 ± 5	7.6 ± 3.8

In contrast, the alloy PTLs show more variation in performance. The polarization curves in Figure 2c show that Inc has similar performance to the Ni PTLs, but HR and SS are significantly more active. The J@2 increases to 2.83 and 3.15 A/cm^2^ and the V@1 decreases to 1.62 and 1.63 V for HR and SS, respectively. To summarize this effect, by switching from the Ni 200 to SS PTL, the J@2 using the same Co_3_O_4_ catalyst increased by a factor of 1.8 and the V@1 decreased by 140 mV. The HFR is slightly higher for Inc and HR at ∼90 mΩ·cm^2^ compared to ∼80 mΩ·cm^2^ for SS and the Ni PTLs, which may indicate increased interfacial contact resistance (Figure 2d). Determining the mechanism by which the alloy PTLs have improved performance is complicated because two properties are varying: morphology and composition.

To investigate the role of morphology, the performance of each PTL was plotted versus the porosity, pore size, and fiber size (Figure 3). As shown by the comparison of the Ni PTLs, where there is no compositional difference, decreased pore size and porosity lead to higher activity. Another useful comparison is between Ni 500 and Inc, which have similar thickness, but vary in pore size, fiber dimension, and composition. Analogous to the comparison of the Ni PTLs, the slightly higher performance of Ni 500 may be attributed to the decrease in average pore size and porosity. The highest performing PTLs have alloy compositions, small fiber dimensions, low porosity, and small pore sizes. To better understand what aspects of performance these PTL properties affect, we next use a voltage breakdown analysis to separate out the impact of the PTL on ohmic, kinetic, *R*_CL_, and mass transport losses.

**Figure 3:**
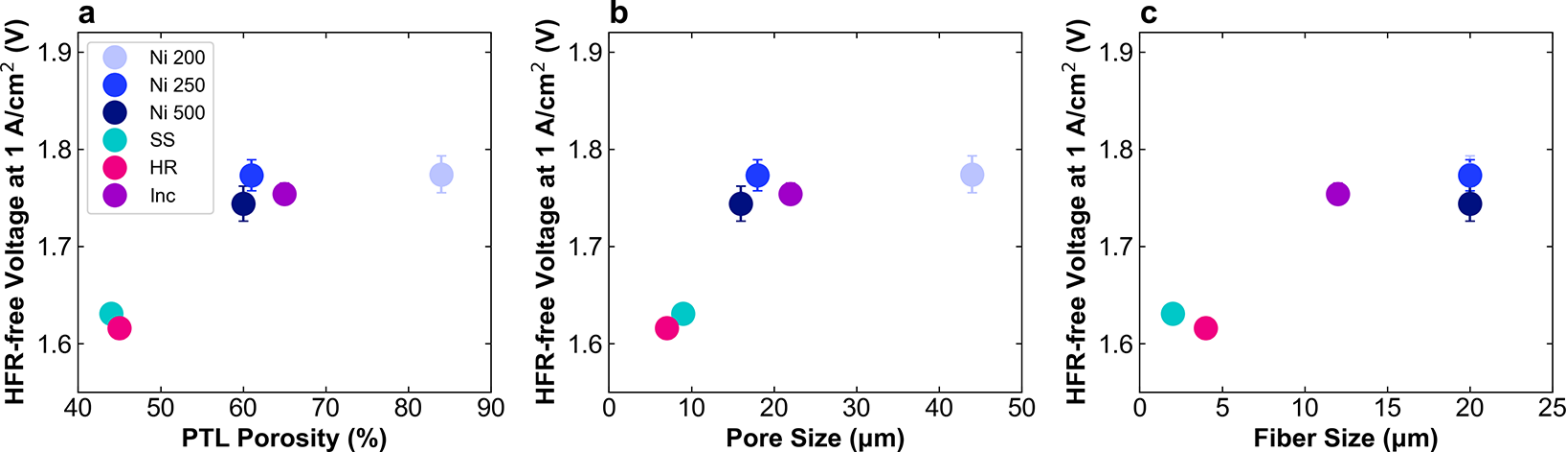
Plots of performance versus morphological properties for Ni 200 (light blue), Ni 250 (blue), and Ni 500 (dark blue), SS (teal), HR (pink), and Inc (purple) PTLs with Co_3_O_4_ catalyst layer. Voltage at 1 A/cm^2^ (HFR-free) versus (a) PTL porosity, (b) pore size, and (c) fiber size. Data are reported in triplicate from separate cell tests.

Figure 4 shows the kinetic and *R*_CL_ portions of the voltage breakdown analysis and a summary of the contribution of the four loss types to the overpotential at 1 A/cm^2^. The full voltage loss breakdown is shown in Figure S4. First, semi-log plots of HFR-free voltage versus current density show the Tafel kinetics (Figure 4a). The Ni and Inc PTLs have only small differences in the Tafel slope and intercept, leading to similar kinetic losses (Figure 4c). In contrast, HR and SS have much lower kinetic losses. For SS, this manifests as a low Tafel slope of 76 mV/dec. HR, however, has a Tafel slope of 94 mV/dec, comparable to Ni or Inc and its lower kinetic losses are instead due to an extremely high Tafel intercept of 120 μA/cm^2^, compared to 4–10 μA/cm^2^ for the other PTLs. These large differences in kinetics depending on the PTL used may be explained by a few different effects, including a decrease in electrode resistances,^51^ an increase in catalyst ECSA, and the catalytic contribution of the PTL. These effects will be discussed in the next sections. The catalyst layer resistances calculated from non-Faradaic EIS indicate that there is a morphological effect on resistances in the catalyst layer, with significantly lower values of *R*_CL_ for HR and SS compared to the Ni and Inc PTLs (Figure 4b). This is likely due to the smaller fiber dimensions of HR and SS, which provide higher material density, improved contact with the catalyst layer, and lower in-plane electronic resistance. Finally, the summary of the voltage loss breakdown shows that the transport losses are lowest for SS, likely due to the hierarchical nature of this PTL, with a dense layer at the catalyst layer interface and a layer with much larger pores that may facilitate bubble transport out of the PTL. Overall, the Ni and Inc PTLs behave similarly, with minimal differences in voltage losses, while HR and SS show substantially higher performance especially due to improved kinetics and mass transport.

**Figure 4:**
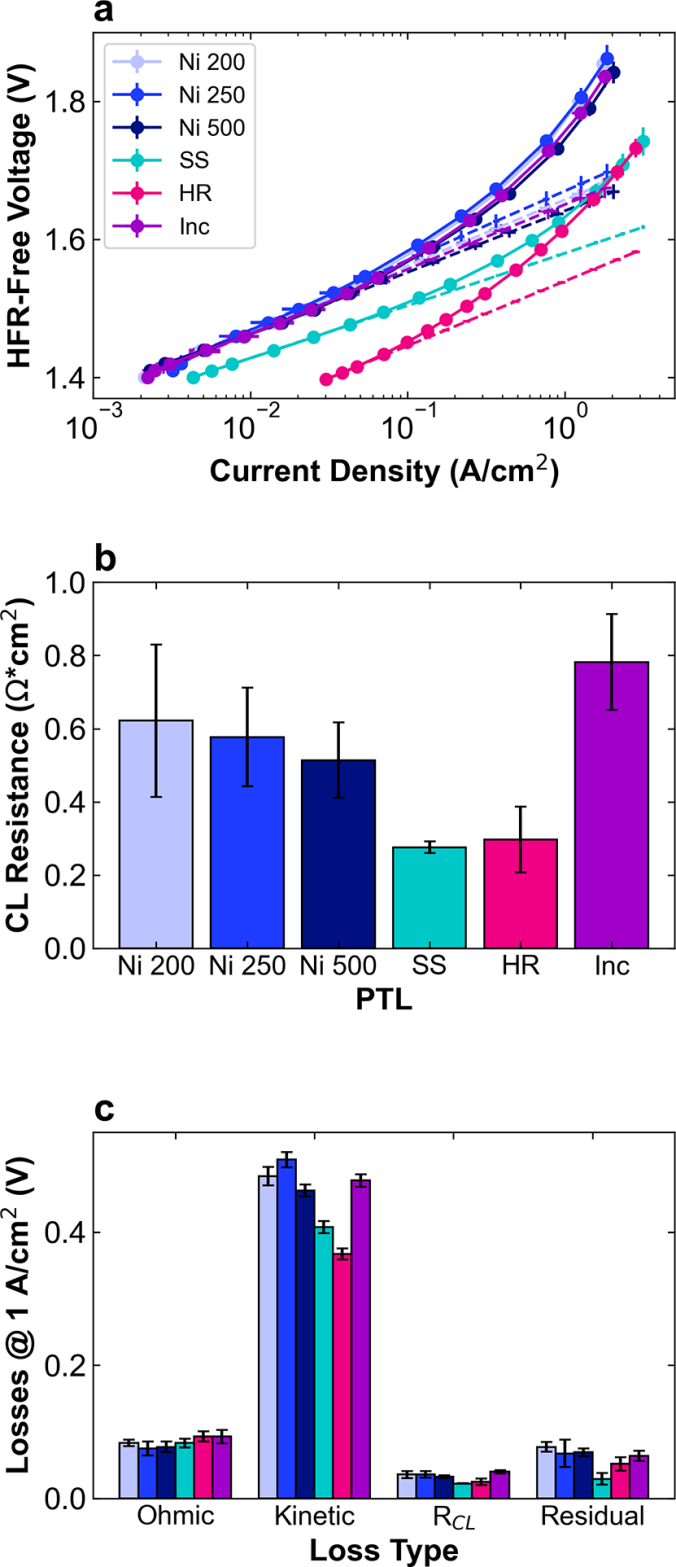
Voltage loss breakdown for Ni 200 (light blue), Ni 250 (blue), Ni 500 (dark blue), SS (teal), HR (pink), and Inc (purple) PTLs with Co_3_O_4_ catalyst layer. (a) Tafel plots with fits shown in dashed lines, (b) catalyst layer resistance values calculated from non-Faradaic EIS at 1.25 V, and (c) summary of ohmic, kinetic, *R*_CL_, and mass transport losses at 1 A/cm^2^. Data are reported in triplicate from separate cell tests.

CVs can provide additional insight into the electrochemical behavior of these anodes, particularly the impact of the PTL. As shown in Figure S5, the Ni and Inc PTLs have similar CV profiles with major features at 1.1/1.0 V and 0.55/0.45 V. The CVs for SS and HR, however, look significantly different, particularly the lack of feature at 0.6 V for SS and the presence of additional features and significant OER current above 1.25 V for HR. While we can conclude that the identity of the PTL has a strong effect on the redox features and capacitance, it is difficult to relate these changes to PTL properties because it is a two-electrode measurement, with contributions from anode and cathode, and there is no fixed reference potential. To address this, the OER activities and CVs of the Ni 250, SS, and HR PTLs were assessed in a half-cell, three-electrode measurement (Figure S6). Although it is an imperfect proxy for ECSA, the double layer capacitance increasing by 2.5x and 60x relative to Ni 250 for HR and SS, respectively, is qualitatively indicative of increased surface area for the alloy PTLs. The redox features in the CVs also show interesting differences. First, for Ni 250, the Ni^2+^/Ni^3+^ redox feature is observed at 1.4/1.3 V vs RHE, which is ∼0.3 V shifted from the single-cell voltage. For the HR PTL, this peak is much larger and is shifted to ∼1.55/1.2 V, likely due to the influence of the Cr, Mo, and Fe dopants. The SS PTL shows broad peaks at ∼1.45 V and 0–0.4 V, which are attributed to Ni^2+^/Ni^3+^ and Fe^2+^/Fe^3+^ transitions, respectively. In the MEA tests, the SS PTL exhibited a broad Ni redox feature, with no Fe feature (Figure S5); this absence may reflect the difference in potential window or a change in Fe behavior in the MEA environment. Finally, the LSVs show the order of OER activity to be HR > SS > Ni 250, although there is an overlap of OER onset with the HR redox feature that makes it difficult to determine the true OER kinetics. Inductively coupled plasma-mass spectrometry (ICP-MS) measurements of the electrolyte after these experiments showed dissolution levels that are negligible compared to the mass of the PTL, but there is preferential loss of both Cr and Mo for the SS and HR PTLs, relative to Fe and Ni, respectively. These experiments show clearly that the PTLs are electrochemically active. Their performance in a MEA without a catalyst layer will be discussed in the next section.

### PTL Performance without Catalyst Layer

To better understand the contribution of the PTL to the AEMWE performance, the PTLs were next studied with no additional catalyst layer (Figure 5). Notably, the bare PTLs demonstrate significant activity. The Ni PTLs show two important trends: the performance is significantly lower without the Co_3_O_4_ catalyst layer and performance now decreases with increasing PTL thickness (Figure 5a). Comparing Ni 200 and 500, the J@2 decreases from 1.55 to 1.22 A/cm^2^ and the V@1 increases from 1.8 to 1.88 V. As shown in Figure 5b, there are no large differences in HFR, with a slightly smaller *R*_CT_ for Ni 200. The alloy PTLs show the same performance trend as they did with the Co_3_O_4_ catalyst, and SS even shows better performance without the catalyst (Figure 5c). These performance metrics are summarized in Table 3.

**Figure 5:**
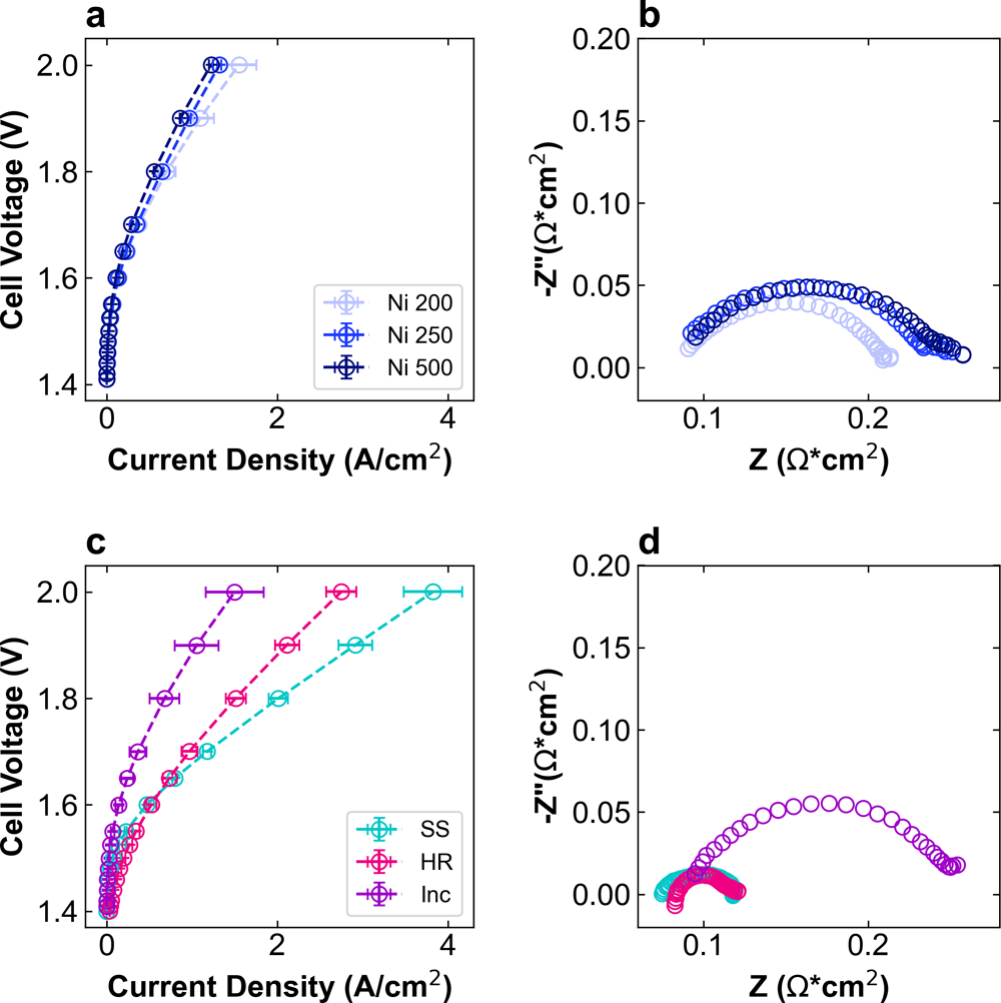
AEMWE performance of various PTLs with no catalyst layer. (a, c) Polarization curves and (b, d) Nyquist EIS plots at 1.9 V for (a, b) Ni 200 (light blue), Ni 250 (blue), and Ni 500 (dark blue) PTLs and for (c, d) SS (teal), HR (pink), and Inc (purple) PTLs. Polarization curves are reported in triplicate from separate cell tests; EIS are from one test chosen as representative of the average.

**Table 3:** Summary of AEMWE Performance for PTLs without a Catalyst Layer

PTL	J@2 (A/cm^2^)	V@1 (V HFR-free)	HFR (Ω·cm^2^)	*R*_CL_ (Ω·cm^2^)	Tafel slope (mV/dec)	*J*_o_ (μA/cm^2^)
Ni 200	1.55 ± 0.21	1.80 ± 0.03	0.080 ± 0.006	1.12 ± 0.18	85 ± 3	4.0 ± 1.5
Ni 250	1.32 ± 0.02	1.85 ± 0.01	0.066 ± 0.011	0.88 ± 0.29	90 ± 2	5.4 ± 1.8
Ni 500	1.22 ± 0.02	1.88 ± 0.00	0.070 ± 0.002	1.06 ± 0.30	90 ± 5	5.6 ± 2.8
SS	3.82 ± 0.34	1.60 ± 0.01	0.075 ± 0.003	0.20 ± 0.08	65 ± 5	1.1 ± 0.8
HR	2.75 ± 0.18	1.61 ± 0.02	0.092 ± 0.005	0.26 ± 0.04	93 ± 3	135 ± 33
Inc	1.50 ± 0.34	1.81 ± 0.05	0.086 ± 0.006	1.02 ± 0.24	85 ± 16	6.4 ± 4.5

Like the Ni PTLs, Inc has worse performance without a catalyst layer and performs similarly to Ni 200. However, HR has very similar activity with and without a catalyst, with J@2 of ∼2.8 A/cm^2^ and V@1 of ∼1.61 V HFR-free. SS shows improved performance in the absence of a catalyst layer, increasing the J@2 from 3.15 to 3.82 A/cm^2^ and reducing the V@1 from 1.63 to 1.60 V. Notably, it also reaches 2 A/cm^2^ at only 1.79 V, meeting the DOE target for performance.^11^ Therefore, depending on the composition and morphology of the PTL, adding a catalyst layer may decrease the performance of the anode. While the EIS results in Figure 5d show similar HFR and *R*_CT_ for HR and SS, these values are significantly higher for Inc. The VBA for the PTLs, analogous to Figure 4, is given in Figure S7. The trends in ohmic, kinetic, *R*_CL_, and transport losses for the different PTLs are similar to and without a catalyst layer. Overall, the variation in performance is larger for the PTL-only electrodes compared to those with catalyst layers; from Ni 500 to SS, the J@2 increases by a factor of 3 and the V@1 decreases by 275 mV. As expected, this indicates that the properties of the PTL affect performance more when there is no catalyst layer. However, plots of performance versus morphological properties show weaker correlations with porosity compared to the case with a catalyst layer, indicating that the composition of the PTL is a more significant factor (Figure S8). Next, we will directly compare the performance of the PTLs with and without catalyst layer to better understand the roles of the PTL and the catalyst layer.

Figure 6 shows a comparison of the AEMWE performance of three representative PTLs with and without the Co_3_O_4_ catalyst layer. Comparisons for all PTLs are given in Figures S9 and S10. As discussed previously, these represent three different effects of catalyst layer addition: the performances are better, worse, and unchanged upon the addition of a catalyst layer for Ni 250, SS, and HR, respectively. Although overall performance improves for Ni 250, the apparent kinetics are slower with the addition of Co_3_O_4_, with the Tafel slope increasing from 90 to 100 mV/dec (Figure 6a­(ii)). The HFR is also slightly higher with Co_3_O_4_, but the *R*_CL_ is decreased (Figure 6a­(iii)). The voltage loss breakdown analysis shows that the most significant effects are increased kinetic losses and decreased residual or mass transport losses with the addition of Co_3_O_4_ (Figure 6 (iv)). These results are unexpected but illuminating. First, this means that the kinetics measured in the MEA do not align with intrinsic activity in RDE, as undoped Ni has been found to be a poor OER catalyst.^52^ Second, the improvement in mass transport indicates that the catalyst layer may facilitate improved transport of water, KOH, and/or gaseous products to/from active sites. Ionic transport is the most likely explanation: with a catalyst layer, more catalyst sites are concentrated near the membrane, and the ionomer network may further improve ionic conductivity to these sites. Without a catalyst layer, catalyst sites are distributed throughout the PTL, requiring OH^–^ transport over longer distances. Thus, while these PTL active sites show high activity in the kinetic regime, there is a significant penalty for ionic transport, which results in overall lower performance for the bare Ni 250 electrode. CVs show that the prominent Ni redox features at ∼1.1 V are smaller when catalyst is present, indicating some “blocking” of PTL sites (Figure 6a­(v)). Nevertheless, many of the features of the CV correspond to the PTL, serving as a reminder that the electrochemical contribution of the “baseline” PTL should not be overlooked.

**Figure 6:**
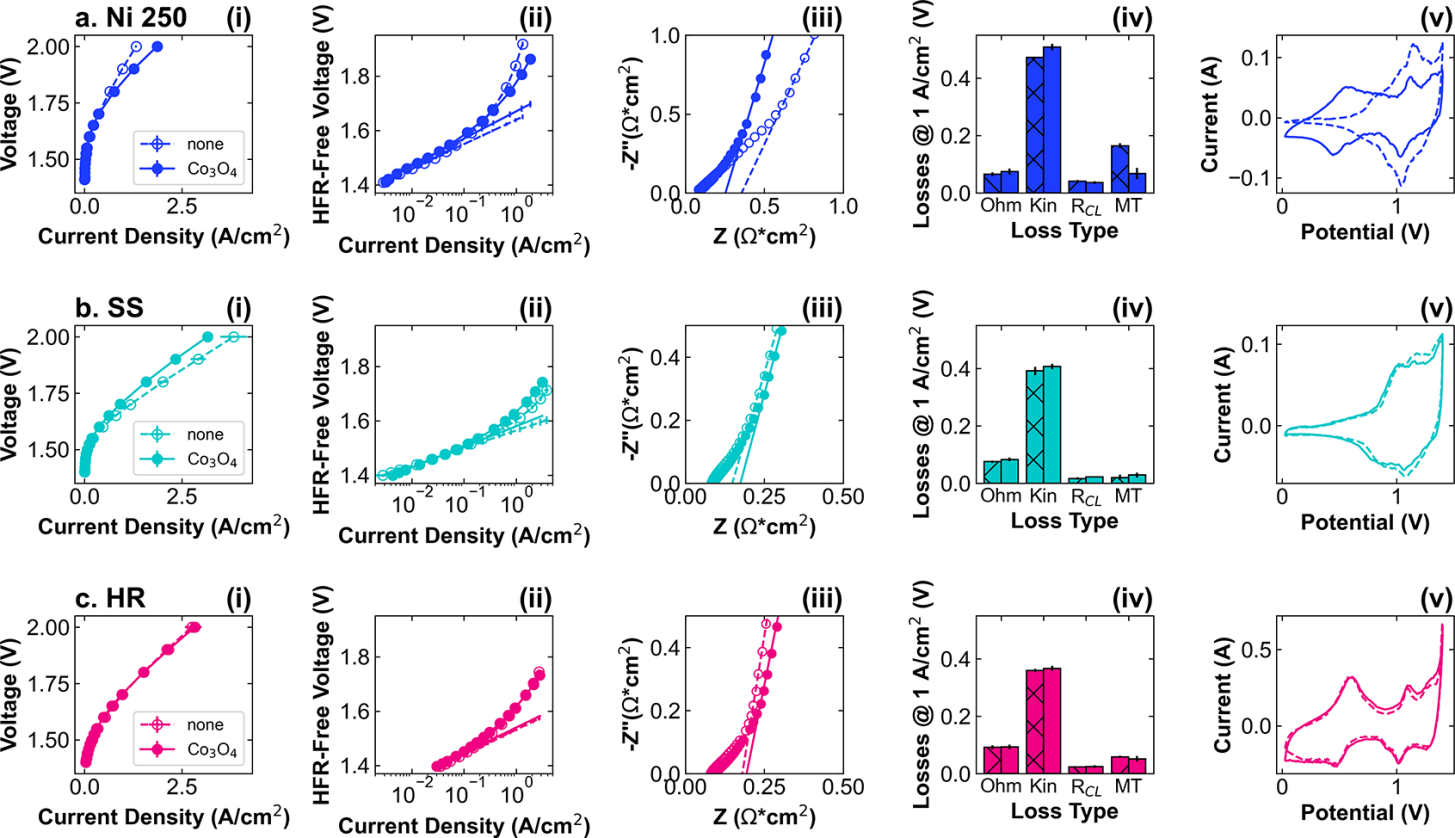
Comparison of AEMWE performance for PTLs with and without Co_3_O_4_ catalyst layer. (i) Polarization curves, (ii) Tafel plots, (iii) Nyquist plot of non-Faradaic EIS at 1.3 V, (iv) VBA summary at 1 A/cm^2^, and (v) CVs at 100 mV/s for (a) Ni 250 (blue), (b) SS (teal), and (c) HR (pink). Tests without Co_3_O_4_ are represented with dashed lines, open symbols, and hatched bars, while tests with Co_3_O_4_ use solid lines, filled symbols, and solid bars. AEMWE performance data are reported in triplicate from separate cell tests; EIS and CVs are representative of the average.

The electrochemical contributions of the PTL are particularly evident for SS, which has a higher performance without the Co_3_O_4_ catalyst layer (Figure 6b). The kinetic losses are slightly lower for the bare SS, due to a decrease in Tafel slope from 75 to 65 mV/dec (Figure 6b­(ii),(iv)), which may indicate higher intrinsic activity for the NiFe active sites in the PTL than for the Co_3_O_4_ catalyst. Like with Ni, there is a reduction in HFR without a catalyst layer, but there is also a decrease in *R*_CL_ (Figure 6b­(iii)). In contrast to the Ni PTL, the lower *R*_CL_ value likely reflects the increased material density in the top layer of the SS, which facilitates electronic transport and increases the number of active sites at the membrane interface, likely decreasing the distance over which OH^–^ must be transported. The mass transport losses are also lower for the bare PTL. The CVs show that redox features are less pronounced for SS, perhaps due to the low Ni content and varied Ni–Fe interactions that impact the Ni^2+^/Ni^3+^ redox potential, but the profile is very similar to and without the Co_3_O_4_ catalyst (Figure 6b­(v)).

Finally, the overall performance and voltage loss breakdown of the HR PTL vary only trivially when the Co_3_O_4_ catalyst layer is added (Figure 6c). As shown in the Tafel plot, there is no difference in Tafel slope, with a slight reduction of the Tafel intercept for the catalyst-coated PTL. The catalyst-coated PTL also demonstrates slightly higher HFR and *R*_CL_, which may indicate that the catalyst is increasing through-plane electronic and/or ionic resistances. There are minimal differences to the CV profile, indicating that the PTL dominates the electrochemically active surface area. Notably, the capacitance and redox features are much larger for HR than the other PTLs, corresponding well to the small pore size and larger expected surface area. Finally, the voltage loss summary indicates that the catalyst has only minor impacts. Taken together, these results suggest that the HR PTL is the active material, and its intrinsic activity and surface area are large enough that its performance is largely unaffected by the presence of this catalyst layer. To better understand the generalizability of this finding, the HR PTL was tested with a higher loading of Co_3_O_4_, as well as with a more active NiFe-based catalyst. With ∼1.5 mg/cm^2^ loading of Co_3_O_4_, roughly corresponding to an increase in catalyst layer thickness from 5 to 10 μm,^22^ the Tafel intercept decreases further, resulting in larger kinetic losses and small negative effects on the J@2 and V@1 (Figure S11). The capacitance and redox features decrease slightly at the higher loading, indicating that the catalyst layer may decrease performance slightly by blocking the more active PTL sites. The same trend is observed for a ∼1.4 mg/cm^2^ loading of Co_3_O_4_ on SS, where the performance decreases further with higher catalyst loading due to increases in Tafel slope, HFR, and *R*_CL_ (Figure S12). Conversely, a 1 mg/cm^2^ loading of the highly active NiFe catalyst on HR results in higher activity than the bare HR PTL, and the activity of the catalyst is the same on Ni 250 or HR PTL (Figure S13). There is a significant decrease in Tafel slope from 93 to 75 mV/dec with the addition of the catalyst, indicating that the active sites have changed, and the catalyst is dominating performance, rather than the PTL. Therefore, the properties of both catalyst layer and PTL will determine the contributions from each material, optimal catalyst design, and overall performance.

### PTL Stability

Beyond activity, the choice of PTL can also significantly affect the durability of the AEMWE device.^53^ While the alloy composition can improve activity, there are concerns about leaching of non-Ni metals like Cr, Fe, and Mo, which can affect the morphology of the PTL and reduce interfacial contact with the catalyst layer, as well as introduce contaminants into the electrolyte. After short-term testing, SEM-EDS was used to assess degradation of the catalyst layer and PTL. The FIB cross-section SEM images with overlaid EDS maps in Figure 7 show how the fibers are affected by testing, as well as the impact of these changes on the catalyst layer. For the Ni 250 PTL, some surface oxidation of the fibers is observed, but there are no significant compositional or morphological changes for the bare or catalyst-coated PTLs (Figure 7a,b). In addition, the catalyst layer is uniform and has consistent coverage of the fiber. In contrast, for the catalyst-coated SS PTL, the overlaid EDS map shows clear compositional changes to the fibers that may also impact the catalyst layer (Figure 7c). Top-down images of the PTL show that there is a uniform catalyst layer with minimal porosity due to the high material density of the SS PTL, but regions with high concentration of Cr and Fe are visible (Figure S14). Individual elemental maps of the PTL show that the bulk PTL remains metallic with minimal composition change relative to the bulk average (Figure S15). Near the interface with the catalyst layer, however, there is significant oxidation and formation of regions of Fe oxide, Cr oxide, and metallic Fe–Cr alloy. This oxidation and segregation of Fe and Cr to the top ∼10 μm of the PTL may be indicative of instability for the portion of the PTL that is participating in OER. Because of this dissolution, gaps have formed between the catalyst layer and PTL and the catalyst layer appears less uniform than on the Ni PTL. In the absence of a catalyst layer, needles of Fe–Cr oxide form out of the top fibers, representing a significant morphological change (Figures 7d and S16). The needles and modified composition are only observed at the top layer of fibers, further indication that the electrochemical activity of the SS is primarily at the membrane-PTL interface. While it is not clear what effect these material changes have on performance, they are a concern for the long-term chemical and mechanical stability of the SS PTL. Finally, the HR PTL shows no large-scale composition or structural change after testing (Figure 7e,f). Top-down images show more visible pores in the Co_3_O_4_ catalyst layer due to the larger fiber-to-fiber distances compared to SS (Figure S14). EDS maps of both the catalyst-coated and bare fibers show a ∼25% decrease in Mo content compared to the bulk average (Figures S17 and S18). To better understand this composition change, the bare HR fibers were imaged at higher magnification (Figure S19). At this scale, clusters of Ni-rich oxide and pits with low Cr and Mo content are visible along the surface of the PTL, indicating that localized segregation and restructuring are also occurring in the HR PTLs. While the mechanism of degradation cannot be determined definitively, it is evident that the oxidation, segregation, and dissolution processes are related for the alloy PTLs.

**Figure 7:**
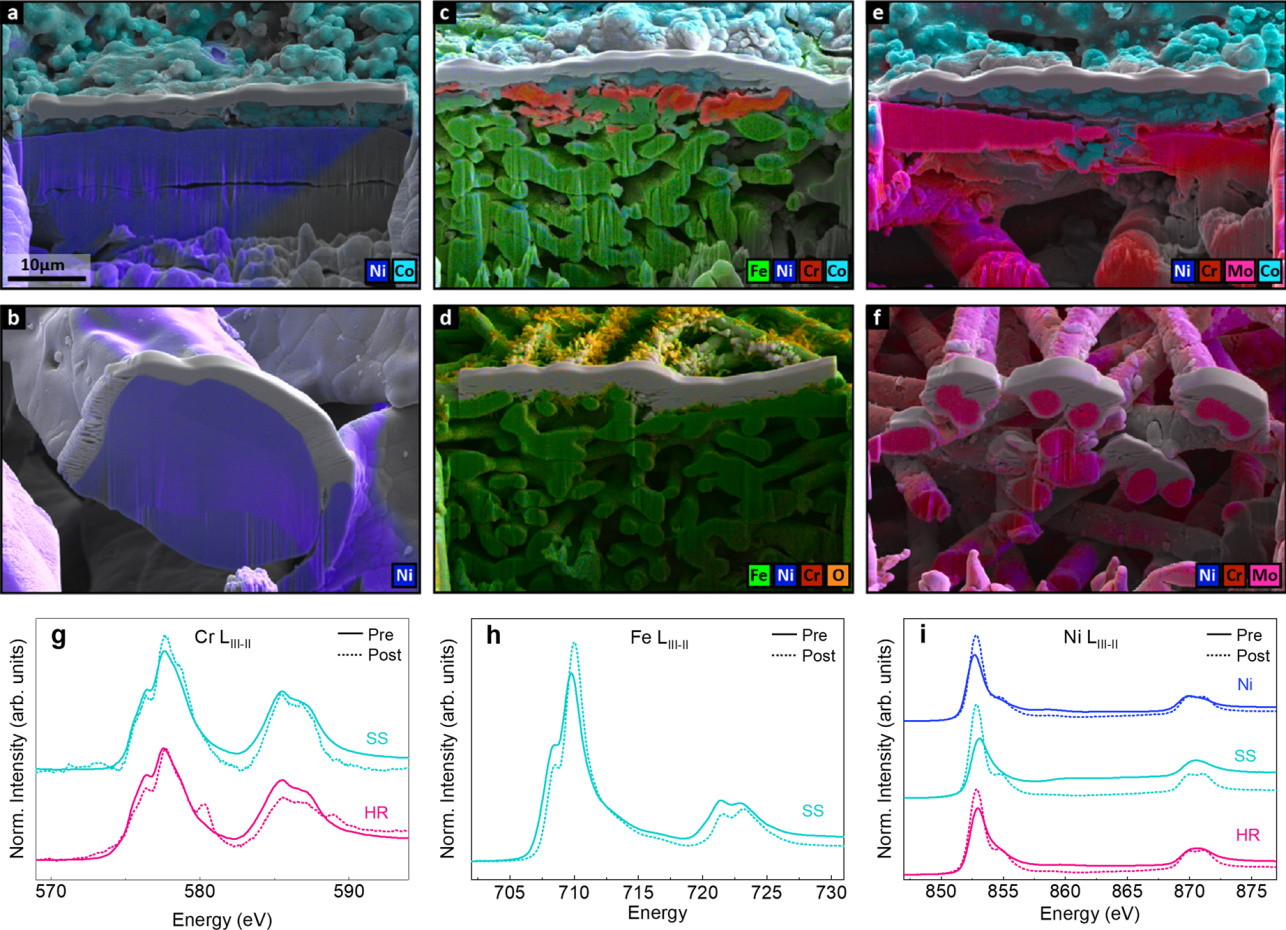
Characterization of Ni 250, SS, and HR PTLs after testing. Overlaid cross-section SEM images and EDS maps of (a) Co_3_O_4_/Ni 250, (b) Ni 250, (c) Co_3_O_4_/SS, (d) SS, (e) Co_3_O_4_/HR, and (f) HR. Elements shown in EDS maps are Ni (blue), Co (light blue), Fe (green), Cr (red), Mo (pink), and O (orange). Individual elemental maps are given in Figures S15–S18. Microscopy experiments are performed after beginning-of-life performance testing, which included a 2 h break-in at 2 V for anodes with Co_3_O_4_ catalyst layer (a, c, e). Pt deposition layers used in FIB cross-sectioning are observed as gray material at the top of each cut. 10 μm scale bar is the same in all images. (g) Cr L_III–II_, (h) Fe L_III–II_, and (i) Ni L_III–II_ edge TEY soft XAS for Ni 250 (blue), SS (teal), and HR (pink) PTLs without a catalyst layer before (solid lines) and after testing (dotted lines).

Next, ex situ XAS was used to further probe these oxidation processes. Because these measurements in TEY mode probe only the top few nanometers, we are able to observe subtle changes that are not evident in the microscopy. Comparing the Cr L_III–II_ spectra for the bare HR PTL, the presence of a high-energy L_III_ peak associated with Cr­(VI) in the posttest spectrum is indicative of an increase in Cr oxidation at the surface (Figure 7g).^54^ This peak is not observed posttest for the bare SS PTL, although both samples exhibit a significant decrease in non-normalized Cr L_III–II_ TEY signal after testing, consistent with a loss of surface of Cr (Figure S2). A shift in intensity towards higher energy is also observed in the Fe L_III–II_ edge after testing for the SS PTL, indicating that the surface Fe is increasing in oxidation (Figure 7h). The ratio of Fe TEY/TFY signal increases when comparing pre- and posttest spectra, suggesting that testing enriches the SS surface in Fe (Figure S2 **).** At the Ni L_III–II_ edge (Figure 7i), the pretest spectra displayed slightly different signals for the three PTLs, showing that there are initial differences in metal character/degree of oxidation. After testing, however, Ni appears similarly oxidized in all PTLs. When the Co_3_O_4_ catalyst layer is used, there are no discernible differences between the Co L_III–II_ spectra for the different PTLs or changes to these spectra due to testing (Figure S20). Additionally, the posttest Cr, Fe, and Ni spectra appear nearly identical to the spectra without a catalyst layer, indicating that the catalyst layer has little impact on the oxidation of the PTL surface.

To understand how these morphology and composition changes may impact electrocatalytic performance, short-term durability tests were conducted for the Ni 250, SS, and HR PTLs without a catalyst layer. Figure 8 shows the results for the 100-h chronopotentiometry (CP) holds at 1 A/cm^2^. For the Ni PTL, which we here consider to be the commercial baseline, there is a degradation rate of 1.2 mV/h from the initial cell voltage of 1.85 V to the final 1.97 V over 100 h (Figure 8a). Compared to this baseline, HR has a higher degradation rate of 1.5 mV/h, while SS is lower at 0.8 mV/h. For all three tests, most of the loss occurs in the first 10 h, with degradation rates of −0.13, −0.34, and 0.63 mV/h from 10 to 100 h for Ni, SS, and HR, respectively. This indicates that Ni and SS are improving over this time, while HR continues to worsen. The rapid initial degradation may be a consequence of the short break-in time used for these tests (2 h), compared to some conditioning procedures for PEMWE that are defined as the time to reach <1% variation in current per hour and can take more than 12 h.^55^ We can also compare the polarization curves and VBA before and after the test. While Ni 250 showed a 120 mV increase in voltage during the CP, in the polarization curve there is a 40 mV *decrease* in V@1 and ∼25% *increase* in J@2 relative to the initial performance (Figure 8b). This recovery is partially due to the exposure to open circuit voltage, which is hypothesized to reverse some of the oxidation processes that lead to rapid voltage increases in the CP. In comparison to the Ni baseline, SS and HR have 34 and 75 mV increases in V@1 and 5 and 25% decreases in J@2, respectively (Figures S21–S23). Thus, the performance losses are only partially recoverable for the alloy PTLs. While XRD shows no change to the bulk metallic structure of the alloy PTLs after testing, some preferential dissolution of Fe, Cr, and Mo, as well as migration of these metals to the cathode is observed (Figure S24). This composition change, as well as the pitting and restructuring observed in microscopy, may prevent full recovery of performance with exposure to open circuit. Unexpectedly, there is also 5–10% loss of Pt mass from the cathode, suggesting that the electrochemical and material stability of the cathode merits further investigation.

**Figure 8:**
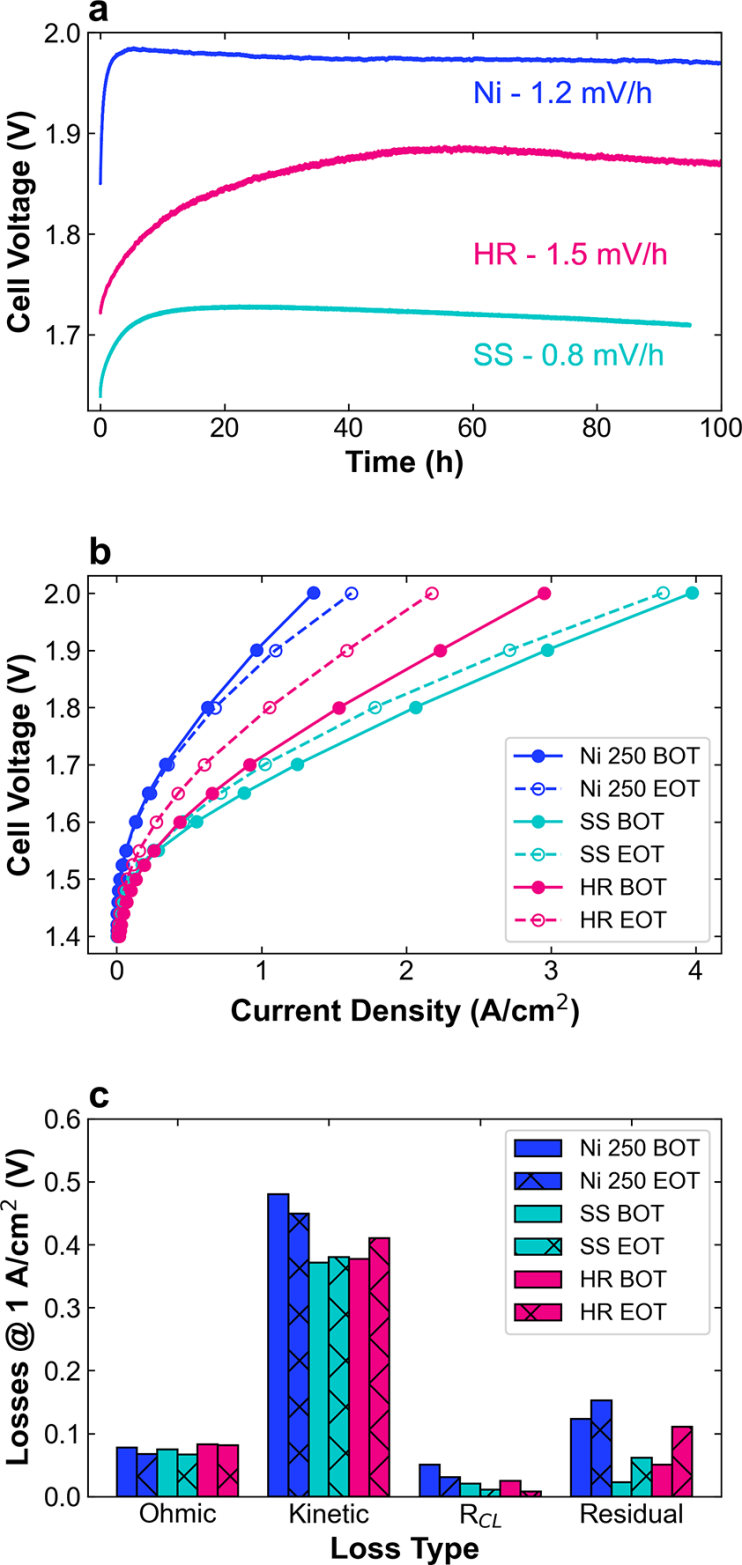
Durability assessment of AEMWE with PTL anodes. (a) 100 h chronopotentiometry measurements for Ni 250 (blue), SS (teal), and HR (pink) PTLs at 1 A/cm^2^. (b) Polarization curves and (c) voltage loss breakdown summary at 1 A/cm^2^ at the beginning of the test (solid lines, filled bars) and the end of the test (dashed lines, hashed bars). The SS test was ended at 95 h due to a potentiostat error.

Finally, the voltage loss breakdown in Figure 8c shows that ohmic and *R*_CL_ losses decrease for each PTL, which is likely related to an increase in KOH concentration during the test, as water is lost to electrolysis and evaporation, that improves ionic conductivity. Mass transport, or residual, losses increase in all cases, which could be due to catalyst layer or PTL compression and restructuring or bubble entrapment; micro-xCT of the electrodes after testing would be valuable in future studies.^56^ The largest difference between the PTLs is the change in kinetic losses. For Ni 250, the kinetic losses decrease and the Tafel slope is lower, perhaps through the formation of oxidized Ni sites with improved intrinsic activity (Figure S21). In contrast, the kinetics worsen for SS with an increased Tafel slope as the surface composition changes (Figure S22). Kinetic losses are also higher for HR after testing with a decrease in the exchange current density, which, when coupled with the observed decrease in the area of redox features in the CVs (Figure S23), may be attributed to a loss of active sites. Despite these trends, the alloy PTLs still have lower kinetic losses than Ni, indicating that the alloy composition remains advantageous. Overall, SS significantly outperforms Ni and HR after the 100 h tests. Despite the larger scale restructuring observed for SS in short-term testing, ICP-MS measurements of the electrolyte indicate lower dissolution rates than for HR. Furthermore, despite its 65% Fe composition, the SS PTL showed a comparable degradation rate to a Ni–Fe based catalyst with only 10% Fe content under identical testing conditions.^22^ The steady decrease in voltage at 1 A/cm^2^ after ∼10 h is a positive indicator for stabilization of the SS surface, but the impacts of restructuring and dopant mobility on longer-term operation are not yet known. Defined standards for conditioning and the “beginning” of the durability measurement are needed to evaluate these tests with respect to the target 4 μV/h degradation rate.^11^ Further investigation of the impacts of PTL restructuring and the mobility of non-Ni metals, as well as the stability of the cathode and membrane, will also be needed.

## Conclusions

In this work, we have investigated the impact of the anode porous transport layer morphology and composition on AEMWE performance. For the catalyst-coated substrate method used here, the PTL morphology, particularly the fiber dimension and pore size, determines the morphology and areal coverage of the catalyst layer. For the three Ni fiber PTLs, a decrease in porosity and pore size is found to have a small, positive effect on performance. The SS and HR PTLs have even lower porosity and smaller pore sizes, resulting in over 100 mV improvement in the HFR-free voltage at 1 A/cm^2^ compared to the best Ni PTL. In addition to the effects on catalyst layer resistance and mass transport, the SS and HR PTLs also lead to significantly improved kinetics through a combination of improved utilization of the Co_3_O_4_ catalyst layer and electrochemical activity from the alloy PTL itself. Without a catalyst layer, the differences in performance between the Ni, SS, and HR PTLs are more drastic due to the high OER activity of the alloy materials, resulting in at least a 200-mV improvement in the HFR-free voltage at 1 A/cm^2^. Without a catalyst layer, SS exceeds the DOE target of 2 A/cm^2^ at 1.8 V. There are concerns, however, about durability of the alloy PTLs and the effects of metal leaching on the membrane and cathode. Using XAS and microscopy, we find that all the PTLs oxidize after testing, but the SS and HR PTLs also undergo segregation, restructuring, and composition changes that may increase contact resistances and decrease the accessible catalyst surface area. However, over 100-h tests at 1 A/cm^2^, the SS PTL exhibits a lower degradation rate than Ni. Although restructuring and dissolution of Fe, Cr, and Mo from these materials may be a concern for long-term operation, the activity and short-term stability are promising.

The PTL significantly affects all aspects of electrochemical behavior and AEMWE performance. The trade-offs with PTL composition, as well as the larger technoeconomic trade-offs of employing supporting electrolyte and catalyst layer-free electrodes, require further study. Future efforts should focus both on the exploration of Ni materials with improved morphologies and on mitigation strategies to improve the durability of alloy materials. Morphological innovations, from simply decreasing porosity and pore sizes to building complex layered structures, offer an opportunity to close the performance gap with the other low temperature electrolysis technologies.

## Supplementary Material



## Notes

Additional catalyst layer characterization and electrochemical measurements (PDF)
