# Supplementary material for: Porous Transport Layers for Anion Exchange Membrane Water Electrolysis: The Impact of Morphology and Composition
Source: ACS Electrochem. 2025 Feb 7;1(6):897–909. doi: 10.1021/acselectrochem.4c00207 (PMC12147155; doi:10.1021/acselectrochem.4c00207)
Supplement: Supplementary file 1 [file ec4c00207_si_001.pdf]

Supporting Information

Porous Transport Layers for Anion Exchange Membrane Water Electrolysis: The Impact of Morphology and Composition

Melissa E. Kreider[1], Ambar Maldonado Santos[1,2], Arielle L. Clauser[3], Matthew E. Sweers[4], Leiming Hu[1], Emily K. Volk[5], Ai-Lin Chan[1], Joshua D. Sugar[3], Shaun M. Alia[1]*

[1] Chemistry and Nanoscience Center, National Renewable Energy Laboratory, Golden, CO, 80401, United States

[2] Department of Chemistry, University of Puerto Rico - Rio Piedras, San Juan, PR, 00931, United States

[3] Sandia National Laboratories, Livermore, CA, 94551, United States

[4] Chemical Sciences and Engineering Division, Argonne National Laboratory, Lemont, IL 60439, United States

[5] Advanced Energy Systems Graduate Program, Colorado School of Mines, Golden, CO, 80401, United States

* Corresponding author: shaun.alia@nrel.gov

Table of Contents

(1) Figures

- **Figure S1.** Cr, Fe, and Ni $L_{III\text{-}II}$ XAS for Ni, SS, and HR PTLs
- **Figure S2.** Ratios of TEY/TFY intensities from Cr, Fe, and Ni $L_{III\text{-}II}$ XAS
- **Figure S3.** Optical microscope images of all 6 PTLs with and without catalyst layer
- **Figure S4.** Voltage breakdown analysis of all 6 PTLs with catalyst layer
- **Figure S5.** CVs of all 6 PTLs with and without catalyst layer
- **Figure S6.** Three-electrode OER LSVs and CVs
- **Figure S7.** Voltage losses and CVs of all 6 PTLs without catalyst layer
- **Figure S8.** Performance versus morphological properties of all 6 PTLs
- **Figure S9.** VBA of all 6 PTLs without catalyst layer
- **Figure S10.** AEMWE performance comparison of all 6 PTLs with and without catalyst layer
- **Figure S11.** AEMWE performance of HR PTL with varying Co_3O_4 loading
- **Figure S12.** AEMWE performance of SS PTL with varying Co_3O_4 loading
- **Figure S13.** AEMWE performance of Ni_8Fe catalyst with different PTLs
- **Figure S14.** Top-down SEM images of SS and HR with and without Co_3O_4 catalyst layer
- **Figure S15.** Single element EDS maps for SS with Co_3O_4 catalyst layer
- **Figure S16.** Single element EDS maps for SS
- **Figure S17.** Single element EDS maps for HR with Co_3O_4 catalyst layer
- **Figure S18.** Single element EDS maps for HR
- **Figure S19.** High magnification SEM images and EDS maps for HR
- **Figure S20.** XAS for PTLs with Co_3O_4 catalyst layer
- **Figure S21.** Voltage loss breakdown analysis of Ni 250 after 100 h test
- **Figure S22.** Voltage loss breakdown analysis of SS after 100 h test
- **Figure S23.** Voltage loss breakdown analysis of HR after 100 h test
- **Figure S24.** Characterization of the Ni, SS, and HR MEAs after 100 h test

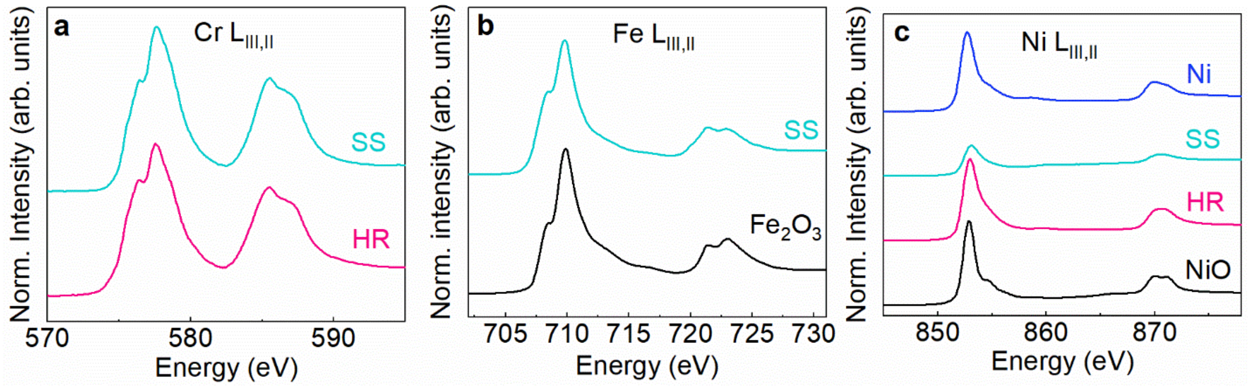

Figure S1. Ex situ soft XAS for (**a**) Cr L_{III-II}, (**b**) Fe L_{III-II}, and (**c**) Ni L_{III-II} edges in TEY mode for the bare Ni (blue), SS (teal), and HR (pink) PTLs prior to testing. Spectra for Fe_2O_3 and NiO (black) powders are included as references.

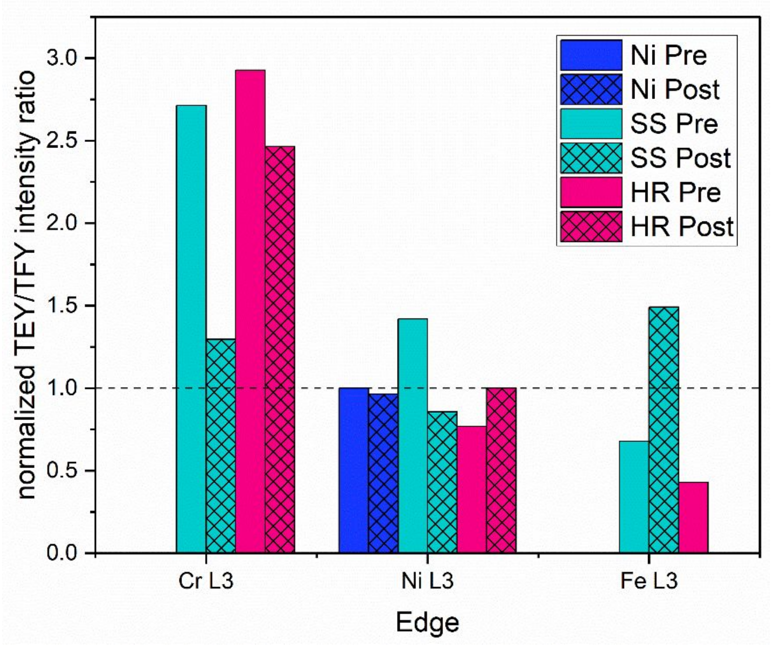

Figure S2. Calculated values of the normalized TEY/TFY signal ratios of the Cr, Fe, and Ni L_{III-II} edges before (solid bars) and after testing (hashed bars) for bare Ni (blue), SS (teal), and HR (pink) PTLs. A ratio above 1 is indicative of relative surface enrichment of that metal.

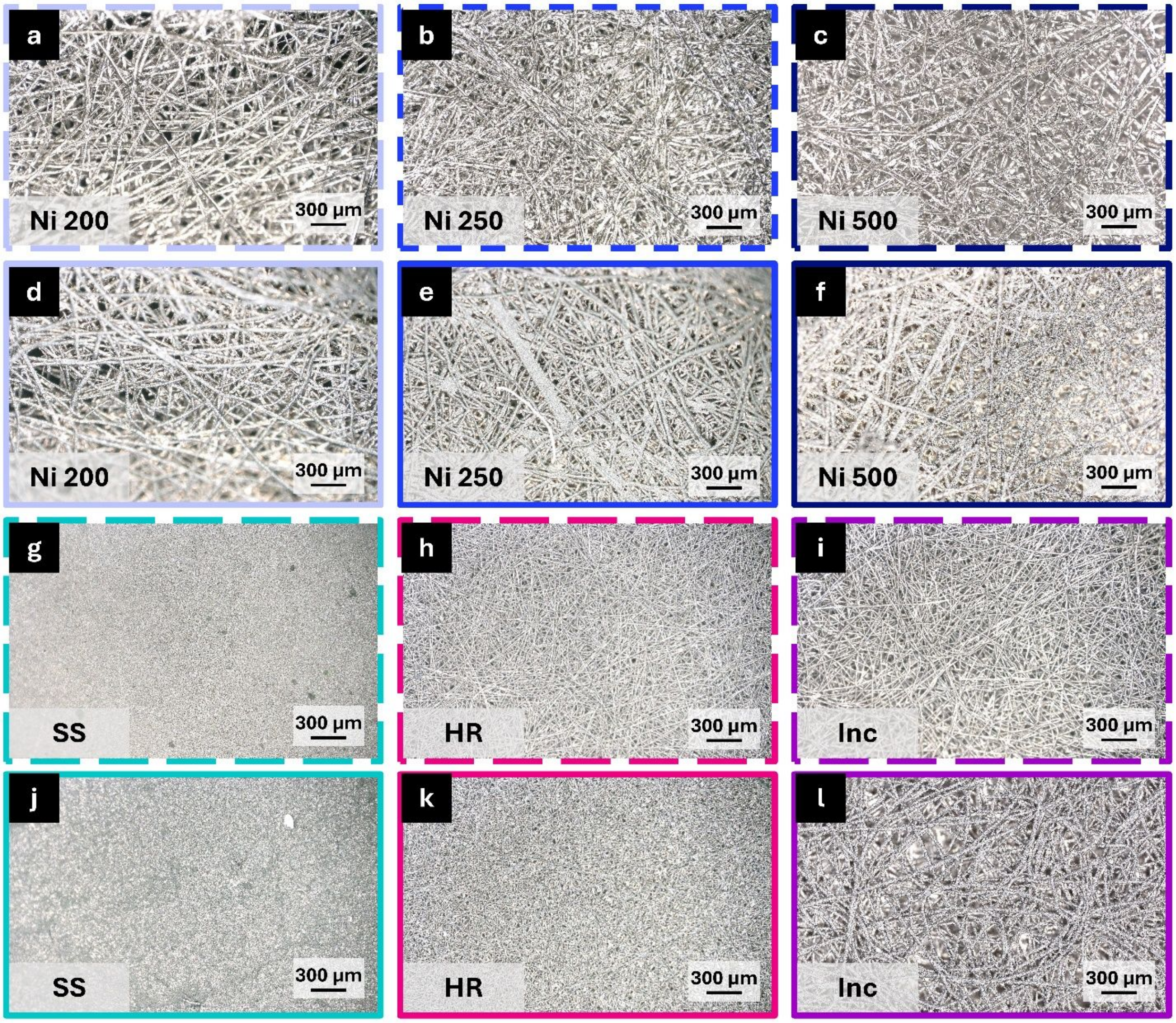

Figure S3. Optical, top-down images of the PTLs before test with no catalyst layer (**A-C**, **G-I**, dashed border) and with a Co_3O_4 catalyst layer (**D-F**, **J-L**, solid border) at 100x magnification. (**A, D**) Ni 200, (**B, E**) Ni 250, (**C, F**) Ni 500, (**G, J**) SS, (**H, K**) HR, and (**I, L**) Inc.

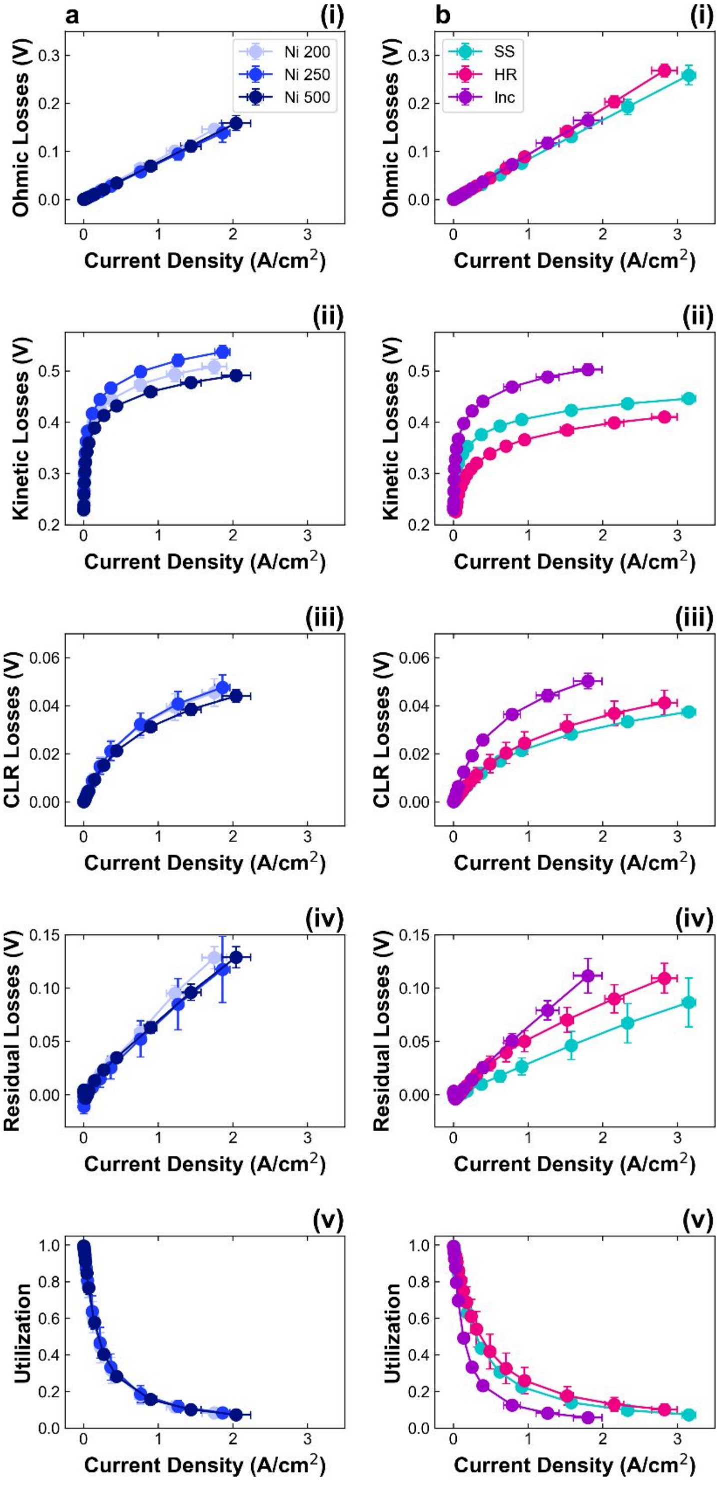

Figure S4. VBA of (**i**) ohmic, (**ii**) kinetic, (**iii**) catalyst layer resistance, and (**iv**) residual (mass transport) losses and (**v**) the corresponding catalyst utilization as a function of current density for Co_3O_4 supported on (**a**) Ni 200, Ni 250, and Ni 500 PTLs, and (**b**) SS, HR, and Inc PTLs.

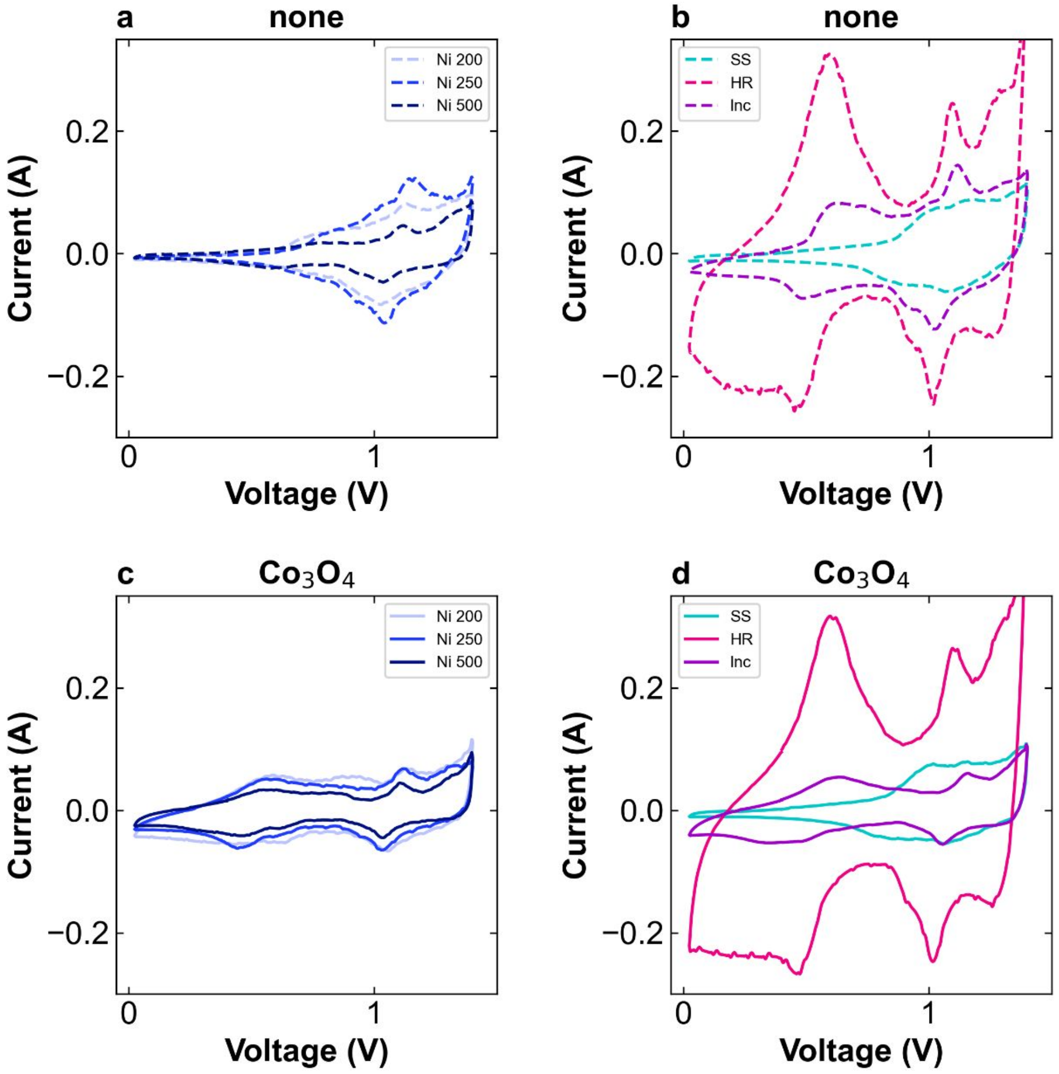

Figure S5. CVs measured in the AEMWE for Ni 200, Ni 250, and Ni 500 PTLs (**a**) without and (**c**) with the Co_3O_4 catalyst layer and SS, HR, and Inc PTLs (**b**) without and (**d**) with the Co_3O_4 catalyst layer at 100 mV/s scan rate from 0.025 to 1.4 V.

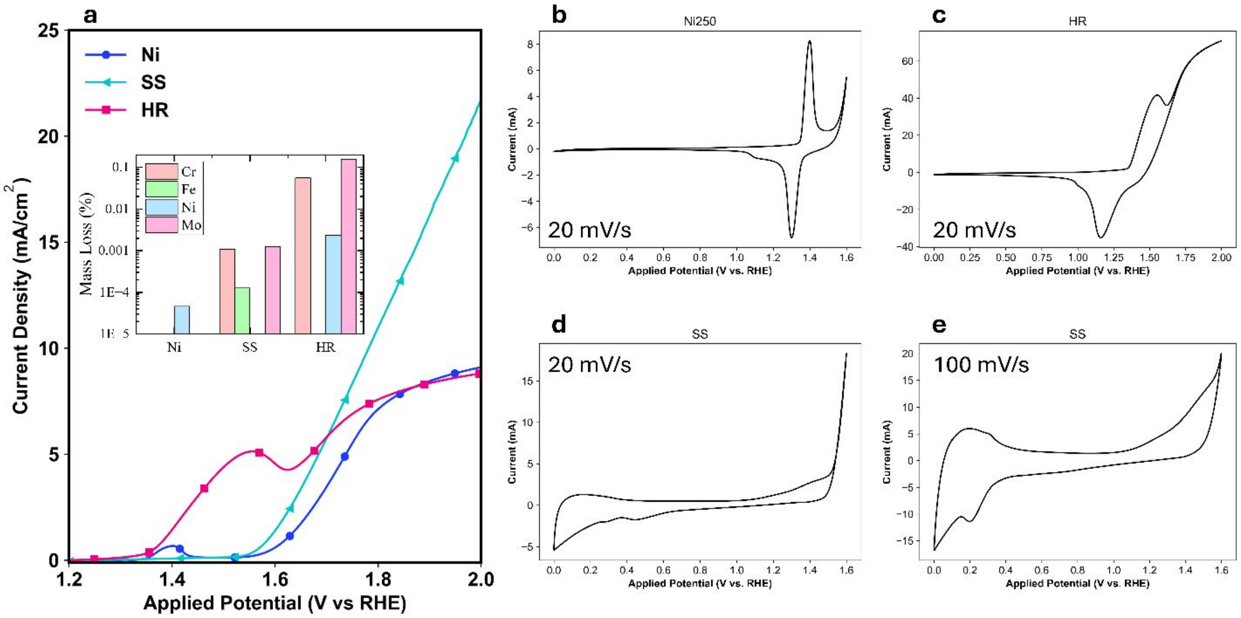

Figure S6. Three-electrode measurements of OER activity and CVs for the Ni 250, HR, and SS PTLs without a catalyst layer. Tested in N_2-purged 1 M KOH with a reversible hydrogen reference electrode and Au counter electrode. (**a**) OER LSVs. Ni 250 and SS were cycled for 10 minutes prior to the measurement; HR was held at 1.8 V for 2 h prior to the measurement to better resolve the redox feature. Inset: mass loss percentage of Cr, Fe, Ni, and Mo as measured by ICP-MS of the electrolyte after testing. CVs for (**b**) Ni 250, (**c**) HR, and (**d**) SS at 20 mV/s and (**e**) SS at 100 mV/s.

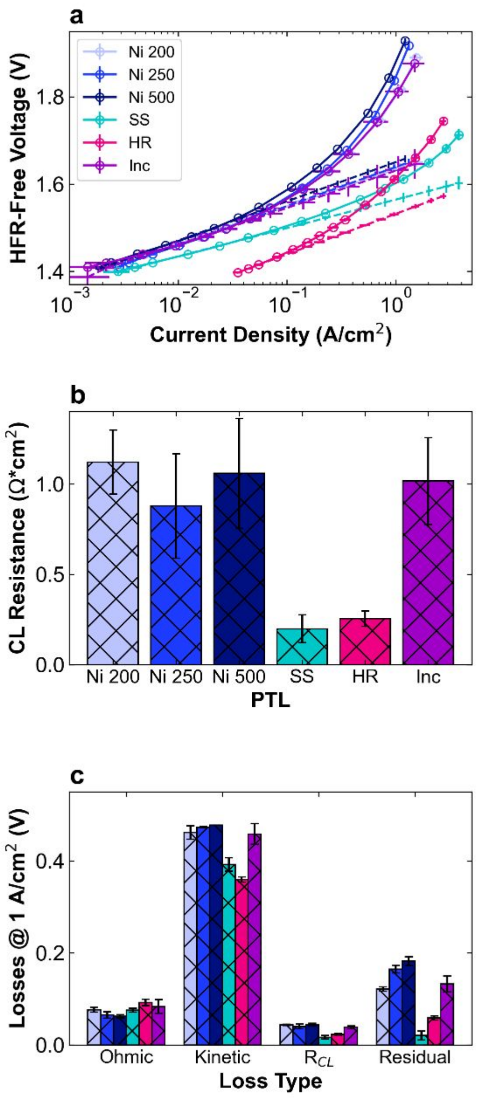

Figure S7. Voltage loss breakdown for Ni 200 (light blue), Ni 250 (blue), Ni 500 (dark blue), SS (teal), HR (pink), and Inc (purple) PTLs without a catalyst layer. (**a**) Tafel plots with fits shown in dashed lines, (**b**) catalyst layer resistance values calculated from non-Faradaic EIS at 1.25 V, and (**c**) summary of ohmic, kinetic, R_{CL}, and mass transport losses at 1 A/cm^2.

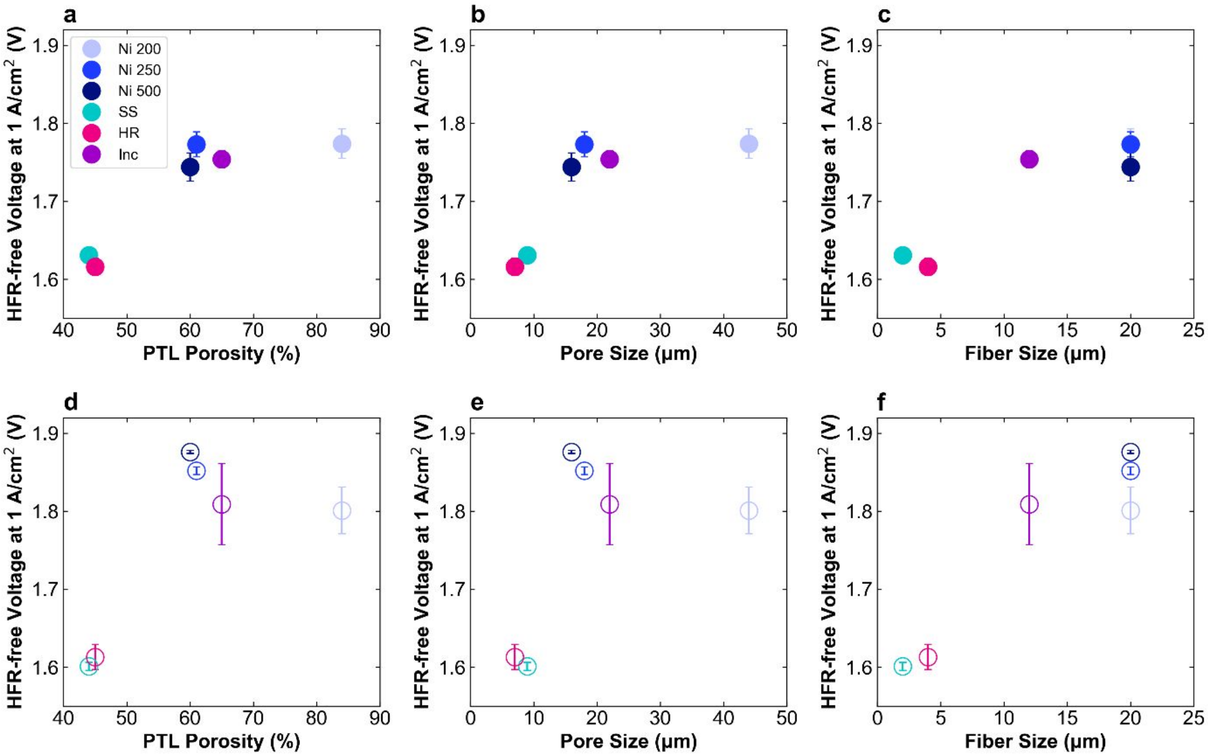

Figure S8. Plots of performance versus morphological properties. Voltage at 1 A/cm^2 (HFR-free) for (**a-c**) PTLs with Co_3O_4 catalyst layer (**Figure 3**) and (**d-f**) without a catalyst layer as a function of (**a, d**) PTL porosity, (**b, e**) pore size, and (**c, f**) fiber size.

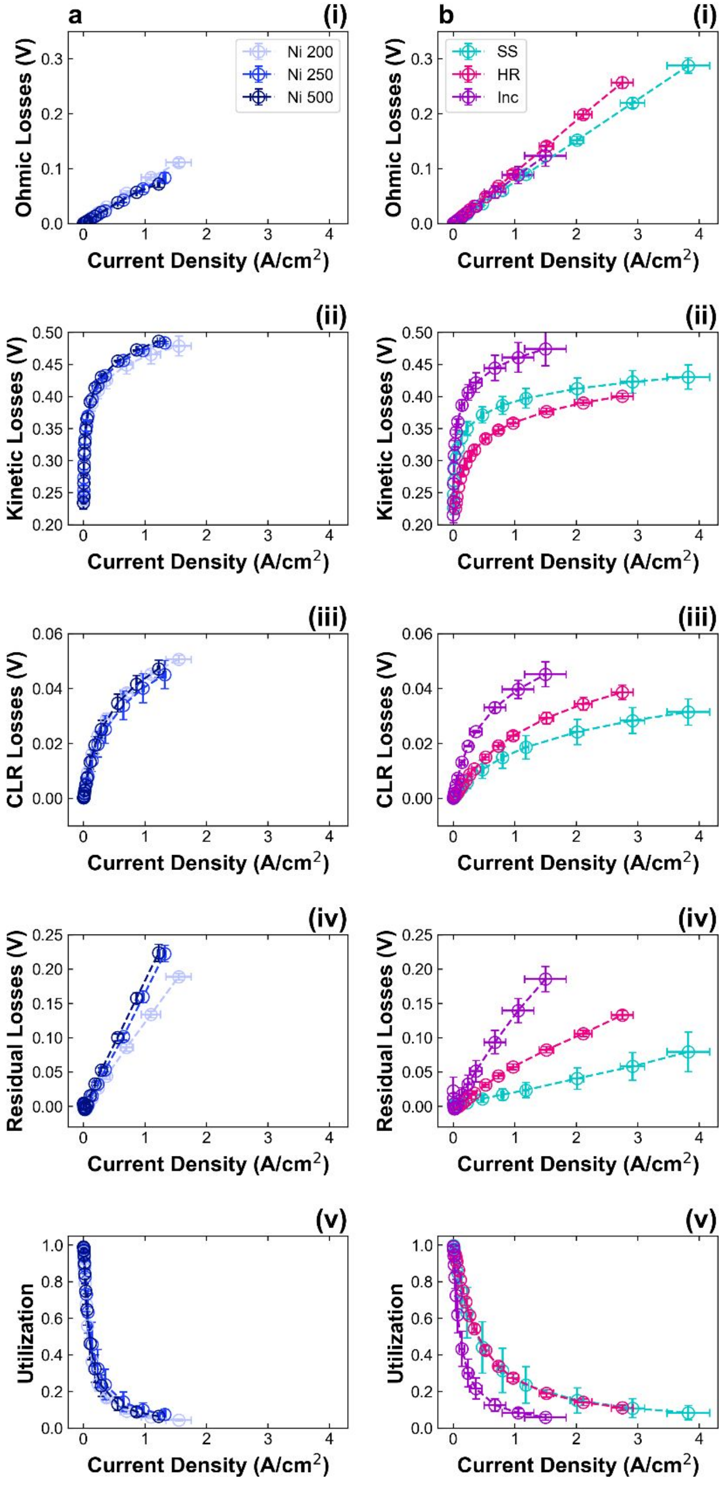

Figure S9. VBA of (**i**) ohmic, (**ii**) kinetic, (**iii**) catalyst layer resistance, and (**iv**) residual (mass transport) losses and (**v**) the corresponding catalyst utilization as a function of current density for (**A**) Ni 200, Ni 250, and Ni 500 PTLs, and (**B**) SS, HR, and Inc PTLs without a catalyst layer.

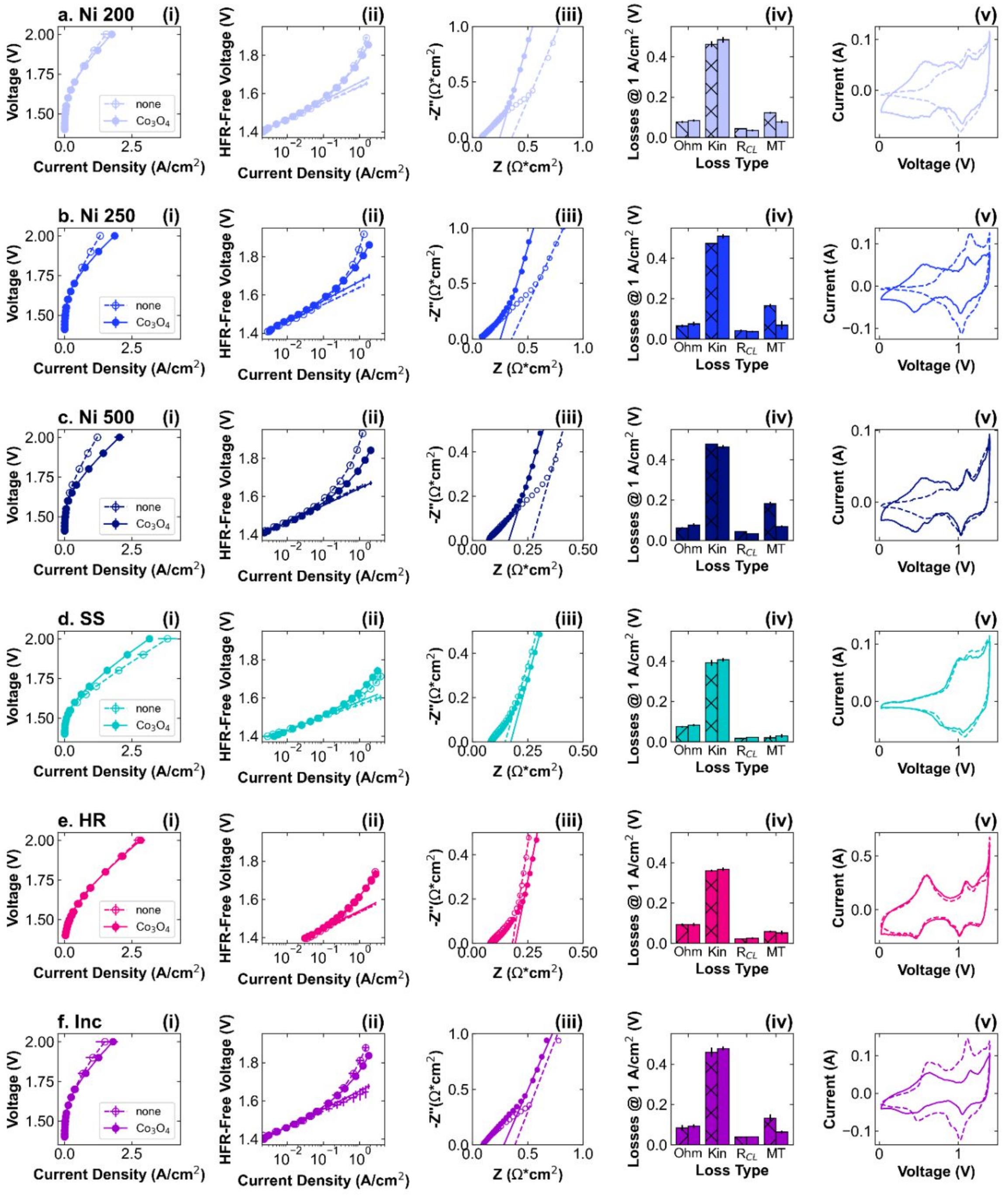

Figure S10. Comparison of AEMWE performance for all 6 PTLs with and without Co_3O_4 catalyst layer. (**i**) Polarization curves, (**ii**) Tafel plots, (**iii**) Nyquist plots of non-Faradaic EIS at 1.3 V, (**iv**) voltage loss breakdown summary at 1 A/cm^2, and (**v**) CVs at 100 mV/s for (**a**) Ni 200 (light blue), (**b**) Ni 250 (blue), (**c**) Ni 500 (dark blue), (**d**) SS (teal), (**e**) HR (pink), and (**f**) Inc (purple). Tests without Co_3O_4 are represented with dashed lines, open circles, and hatched bars, while tests with Co_3O_4 use solid lines, filled circles, and solid bars. AEMWE performance is reported in triplicate; EIS and CVs are representative of average.

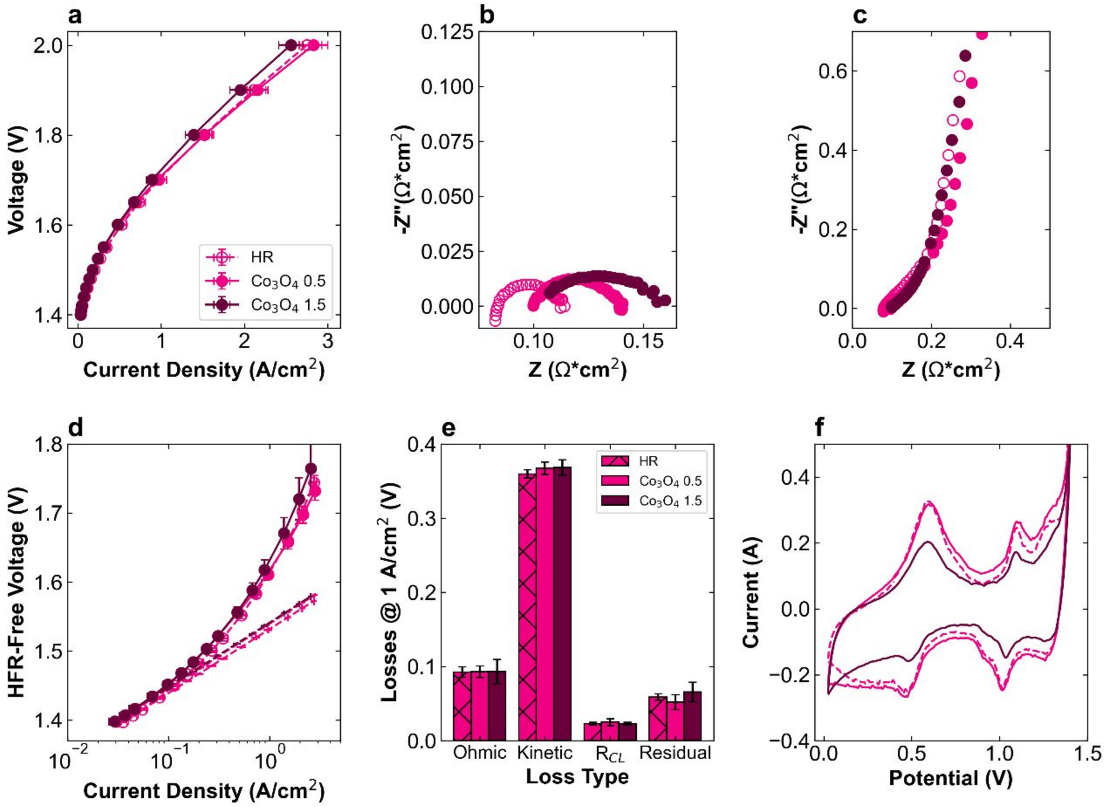

Figure S11. AEMWE performance of HR PTL with varied loadings of Co_3O_4. (**a**) Polarization curves, Nyquist plots of EIS at (**b**) 2 V and (**c**) 1.3 V, (**d**) Tafel plot, (**e**) voltage loss breakdown summary at 1 A/cm^2, and (**f**) CVs at 100 mV/s for 0 (pink, dashed line, open circle), 0.5 (pink, solid line, filled circle), and 1.5 mg/cm^2 (dark pink, solid line, filled circle) loadings of Co_3O_4.

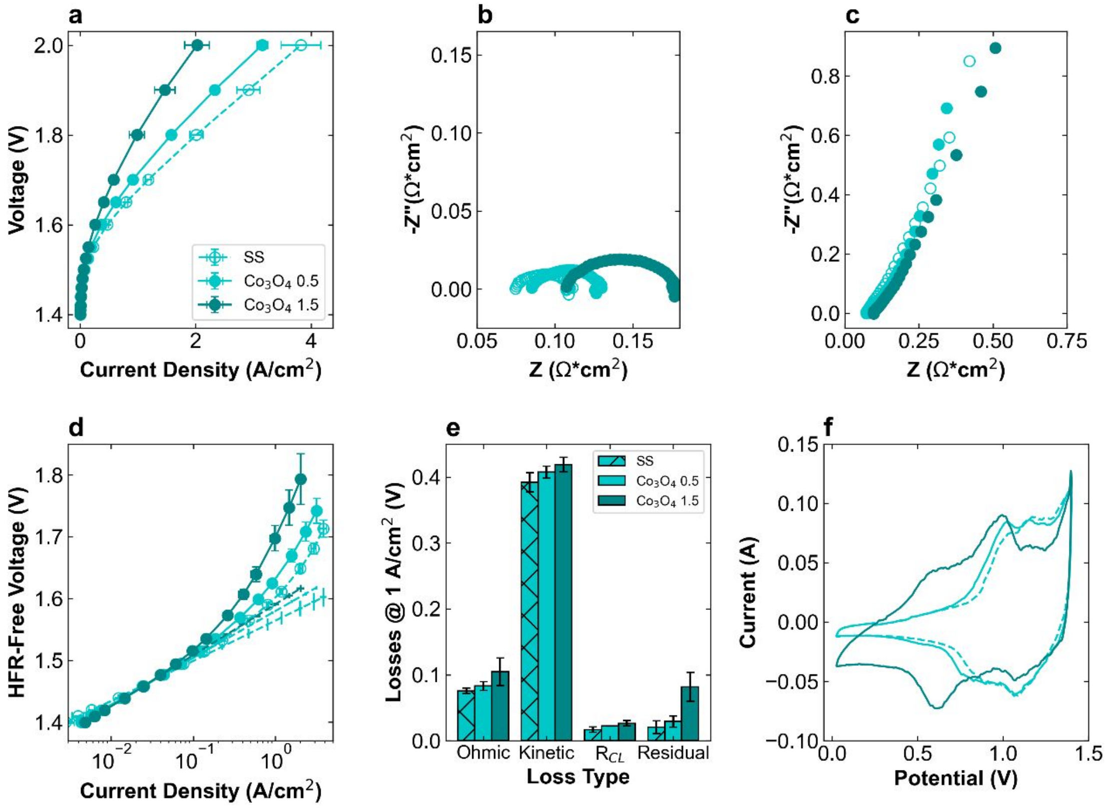

Figure S12. AEMWE performance of SS PTL with varied loadings of Co_3O_4. (**a**) Polarization curves, Nyquist plots of EIS at (**b**) 2 V and (**c**) 1.25 V, (**d**) Tafel plot, (**e**) voltage loss breakdown summary at 1 A/cm^2, and (**f**) CVs at 100 mV/s for 0 (teal, dashed line, open circle), 0.5 (teal, solid line, filled circle), and 1.5 mg/cm^2 (dark teal, solid line, filled circle) loadings of Co_3O_4.

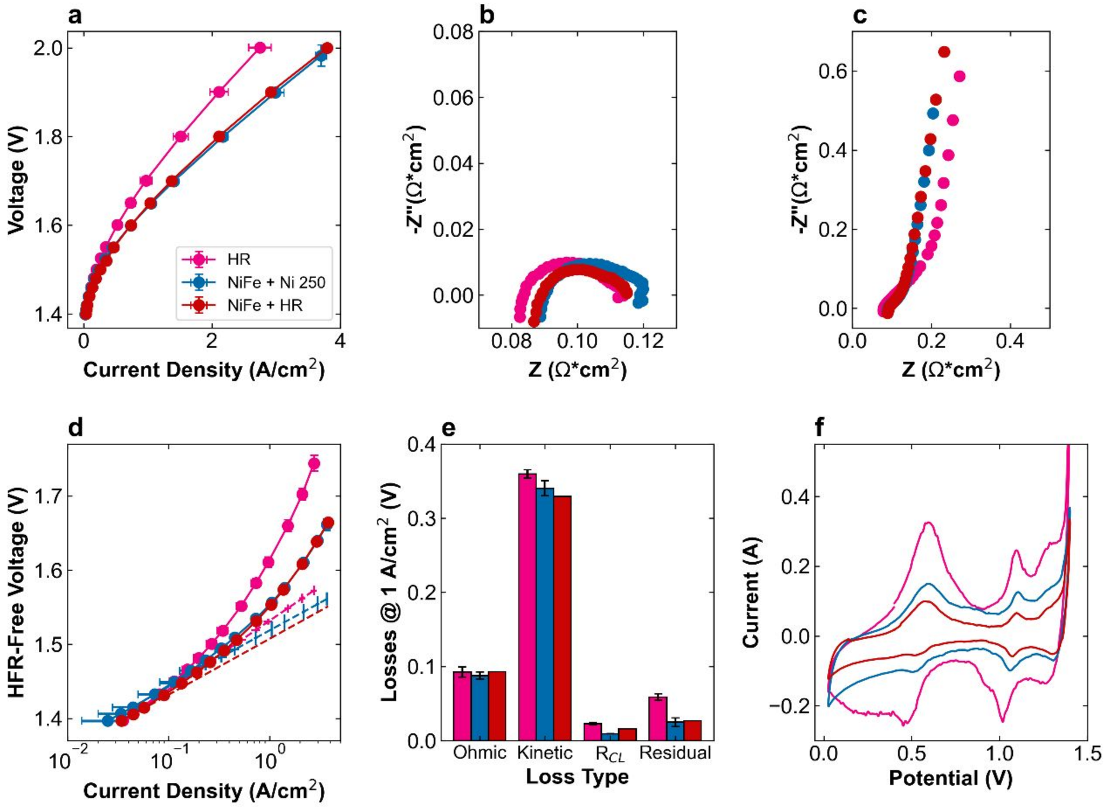

Figure S13. AEMWE performance comparison of HR and a NiFe catalyst supported on Ni 250 or HR. (**a**) Polarization curves, Nyquist plots of EIS at (**b**) 2 V and (**c**) 1.3 V, (**d**) Tafel plot, (**e**) voltage loss breakdown summary at 1 A/cm^2, and (**f**) CVs at 100 mV/s for HR (pink), NiFe on Ni 250 PTL (dark blue), and NiFe on HR PTL (dark red).

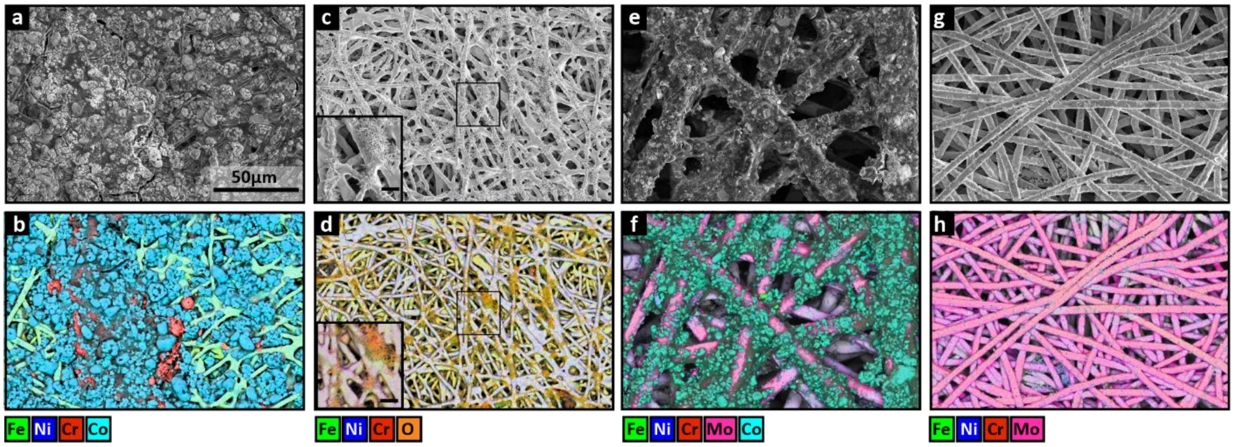

Figure S14. Microscopy of SS and HR PTLs after testing. Top-down SEM images and EDS maps of (**a, b**) Co_3O_4/SS, (**c, d**) SS, (**e, f**) Co_3O_4/HR, and (**g, h**) HR after testing. Elements shown in EDS maps are K (light blue), Cr (pink), Fe (orange), Co (green), Mo (blue), and Ni (yellow).

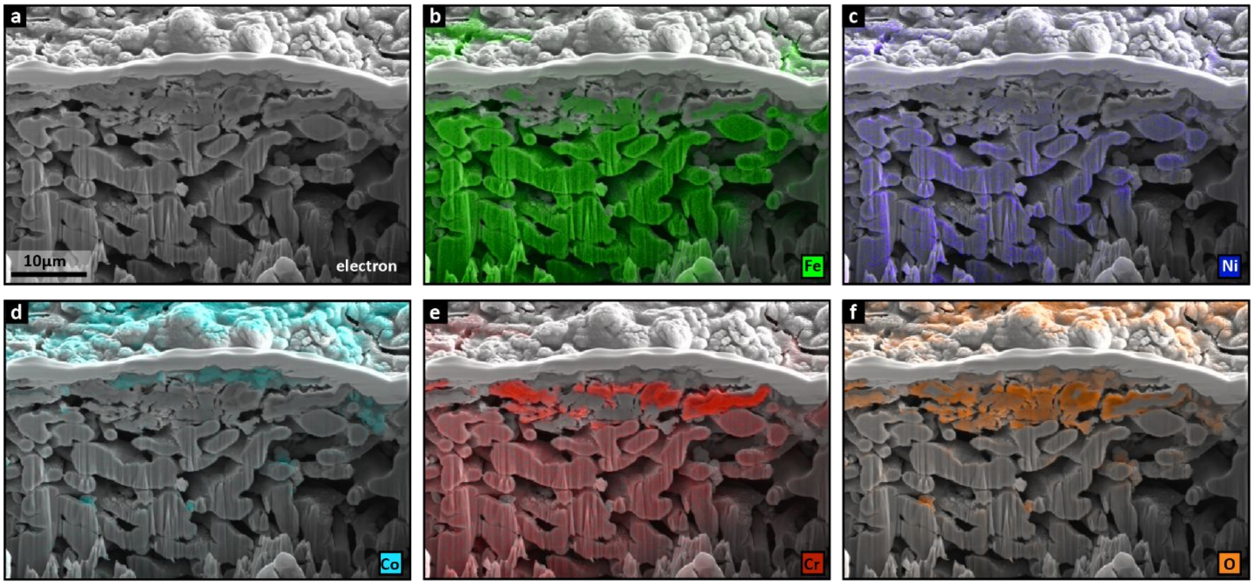

Figure S15. Single element EDS maps of the SS PTL with Co_3O_4 catalyst layer showing (**a**) the FIB cross-section electron image, the electron map overlaid with the (**b**) Fe, (**c**) Ni, (**d**) Co, (**e**) Cr, and (**f**) O EDS signal maps. Individual maps resolve spatially the portions of the cross-section that are oxides that have developed on the PTL and elemental variation throughout the decomposition products, in this case Cr oxides and NiFe oxides.

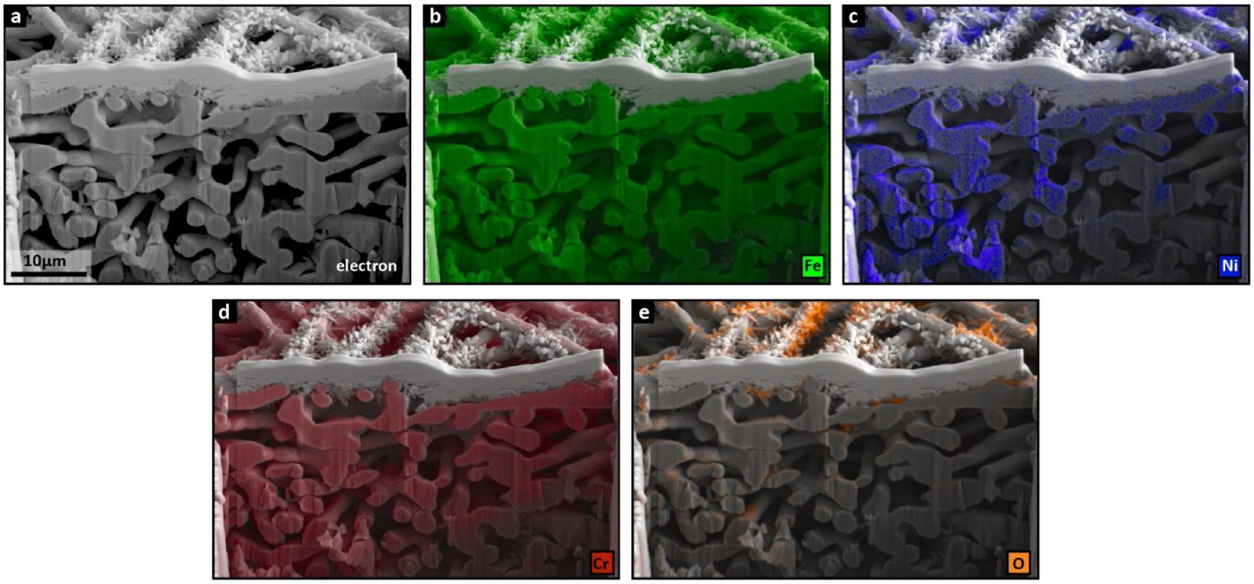

Figure S16. Single element EDS maps of the SS PTL showing (**a**) the FIB cross-section electron image, the electron map overlaid with the (**b**) Fe, (**c**) Ni, (**d**) Cr, and (**e**) O EDS signal maps. The SS PTL with no catalyst layer shows needles of Fe oxide forming on the surface of the PTL, these are easily discernible in **e** the O electron and EDS image.

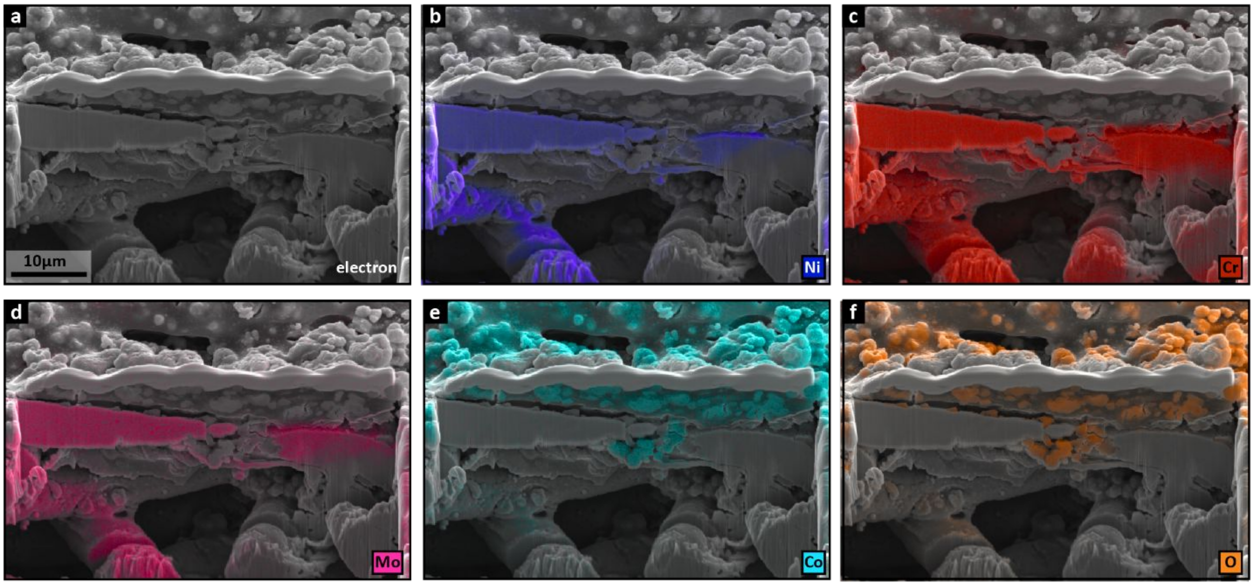

Figure S17. Single element EDS maps of the HR PTL with Co_3O_4 catalyst layer showing (**a**) the FIB cross-section electron image, the electron map overlaid with the (**b**) Ni, (**c**) Cr, (**d**) Mo, (**e**) **Co** and (**f**) O EDS signal maps.

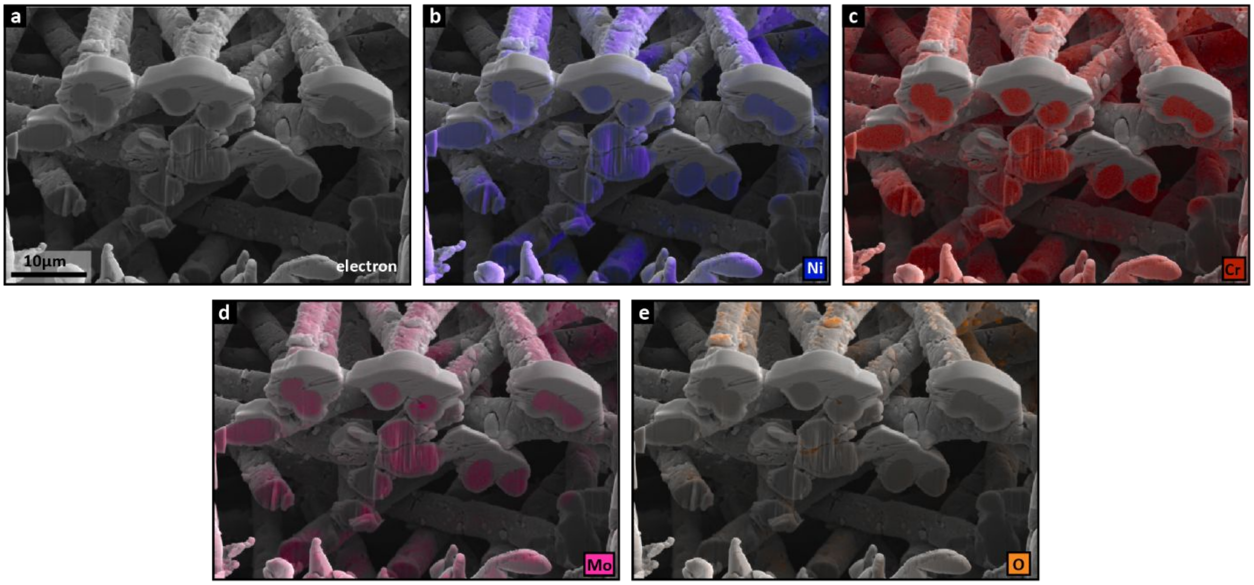

Figure S18. Single element EDS maps of the HR PTL showing (**a**) the FIB cross-section electron image, the electron map overlaid with the (**b**) Ni, (**c**) Cr, (**d**) Mo, (**e**) **Co** and (**f**) O EDS signal maps. At this magnification some oxide layer can be observed forming on the surface of the bare PTL fibers.

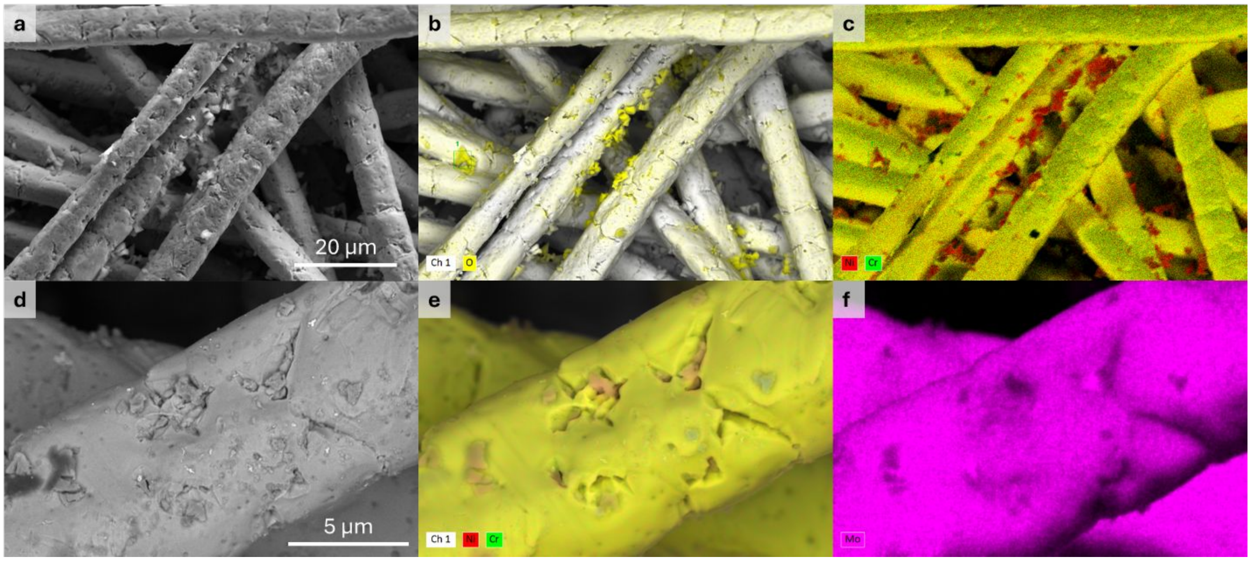

Figure S19. Higher magnification images of HR PTL after testing without a catalyst layer. Top-down SEM images at 2500x (**a**) and 12,000x magnification **(d)** with EDS maps of (**b**) O (yellow), (**c, e**) Ni (red) and Cr (green), and (**f**) Mo (pink).

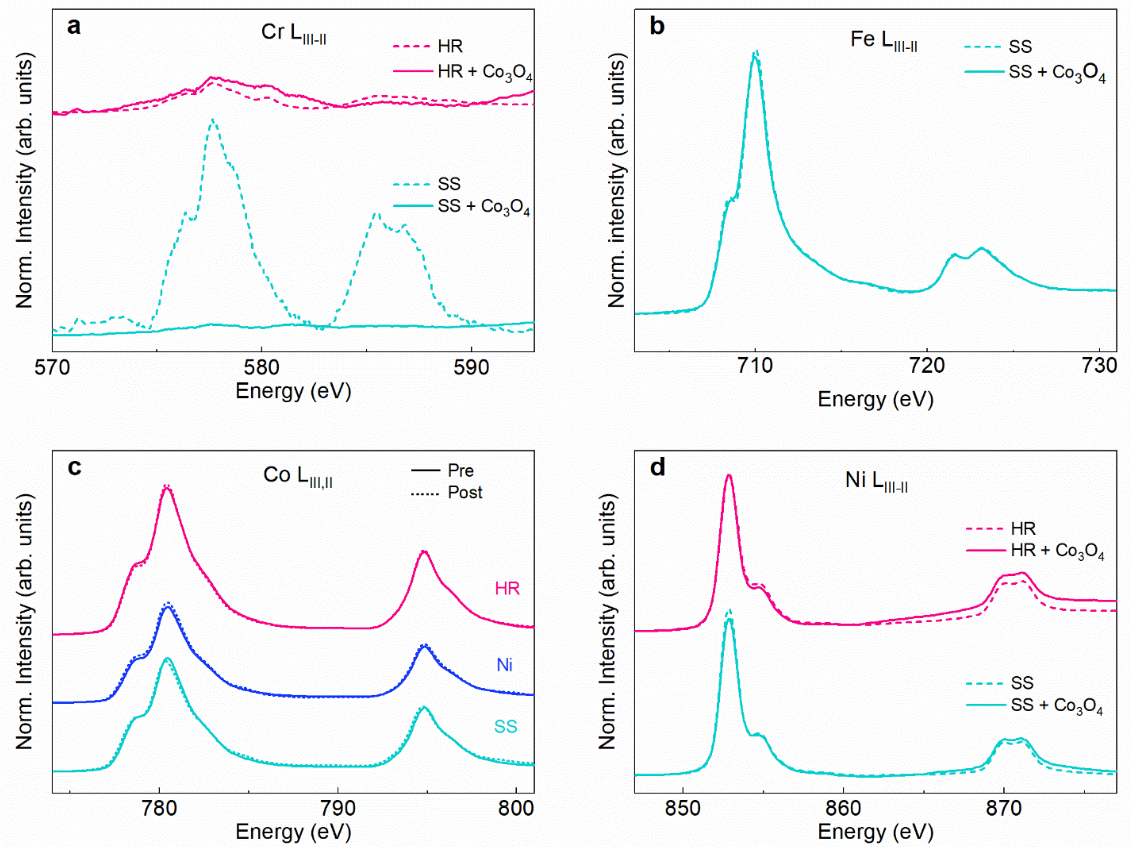

Figure S20. Ex situ soft XAS for (**a**) Cr $L_{III\text{-}II}$, (**b**) Fe $L_{III\text{-}II}$, (**c**) Co $L_{III\text{-}II}$, and (**d**) Ni $L_{III\text{-}II}$ edges in TEY mode for the Ni (blue), SS (teal), and HR (pink) PTLs with (solid lines) and without Co_3O_4 (dashed lines) catalyst layers prior to testing.

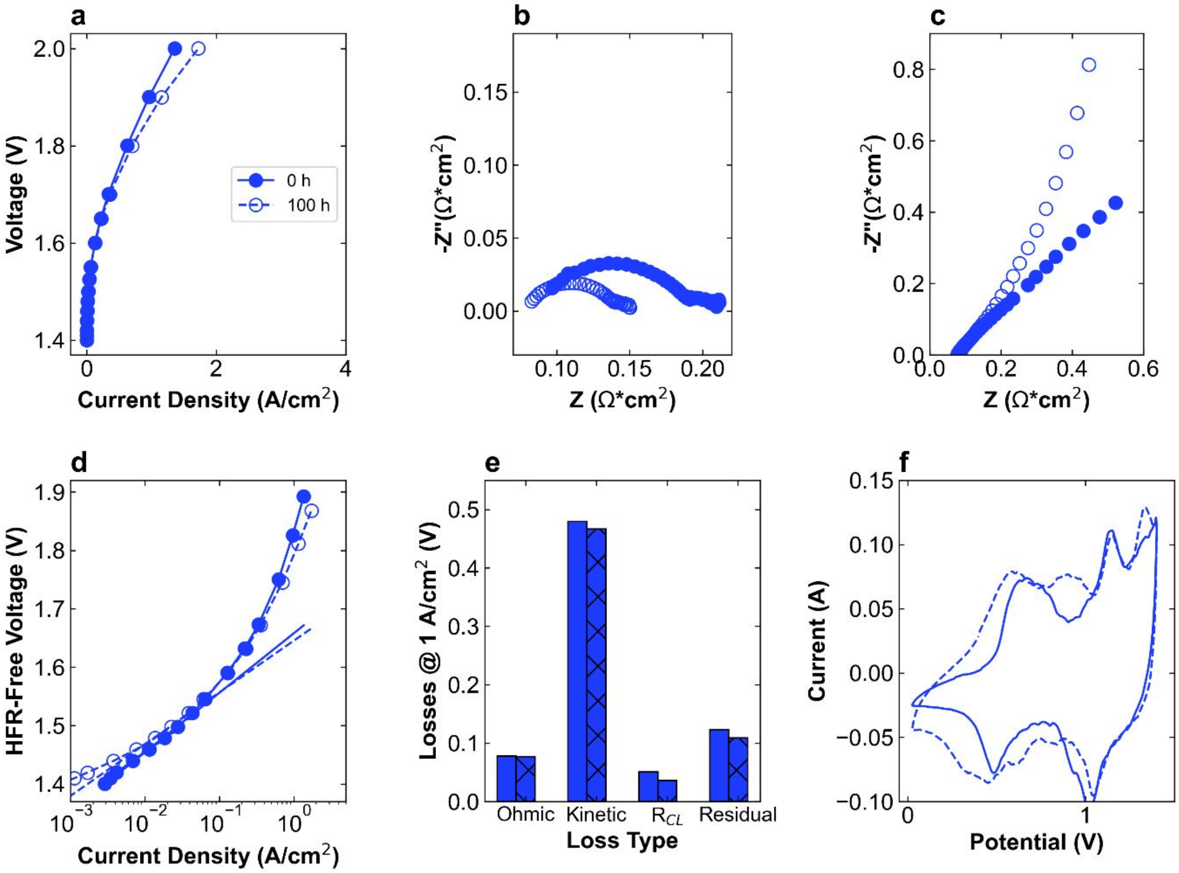

Figure S21. AEMWE performance comparison of Ni 250 before and after the durability test in **Figure 7**. (**a**) Polarization curves, Nyquist plots of EIS at (**b**) 2 V and (**c**) 1.3 V, (**d**) Tafel plot, (**e**) voltage loss breakdown summary at 1 A/cm^2, and (**f**) CVs at 100 mV/s before (solid line, filled circle) and after testing (dashed line, open circle).

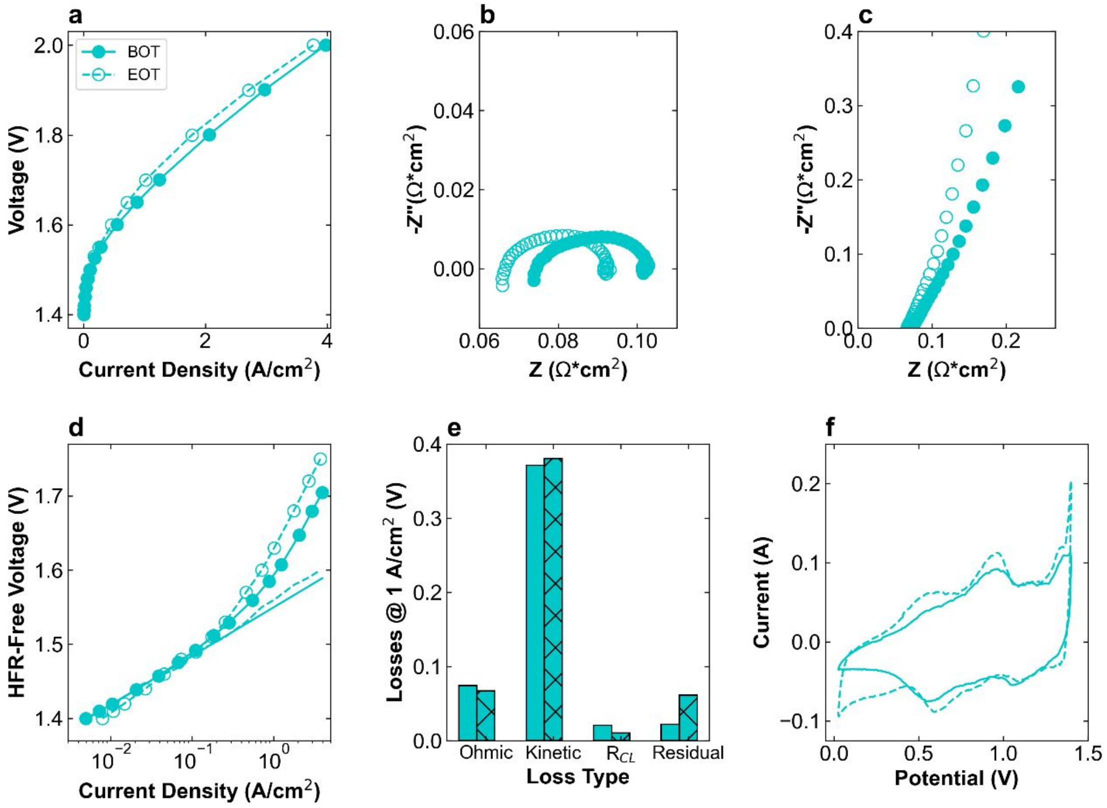

Figure S22. AEMWE performance comparison of SS before and after the durability test in **Figure 7**. (**a**) Polarization curves, Nyquist plots of EIS at (**b**) 2 V and (**c**) 1.3 V, (**d**) Tafel plot, (**e**) voltage loss breakdown summary at 1 A/cm^2, and (**f**) CVs at 100 mV/s before (solid line, filled circle) and after testing (dashed line, open circle).

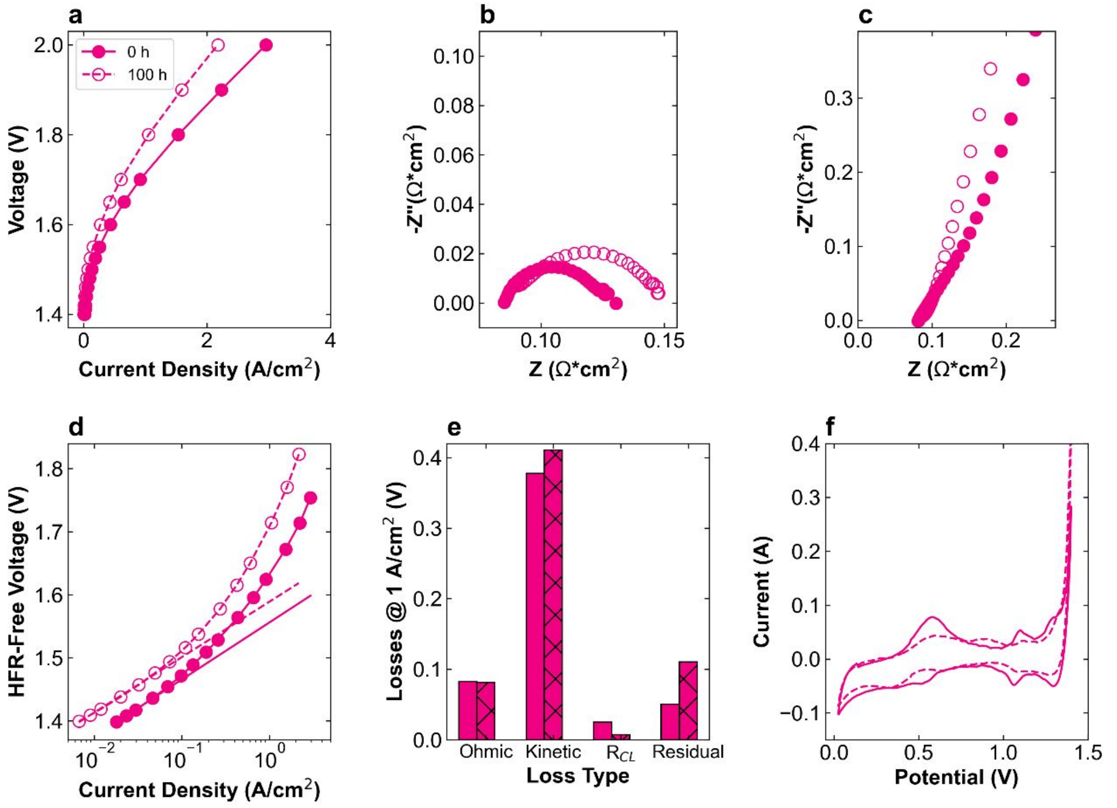

Figure S23. AEMWE performance comparison of HR before and after the durability test in **Figure 7**. (**a**) Polarization curves, Nyquist plots of EIS at (**b**) 2 V and (**c**) 1.3 V, (**d**) Tafel plot, (**e**) voltage loss breakdown summary at 1 A/cm^2, and (**f**) CVs at 100 mV/s before (solid line, filled circle) and after testing (dashed line, open circle).

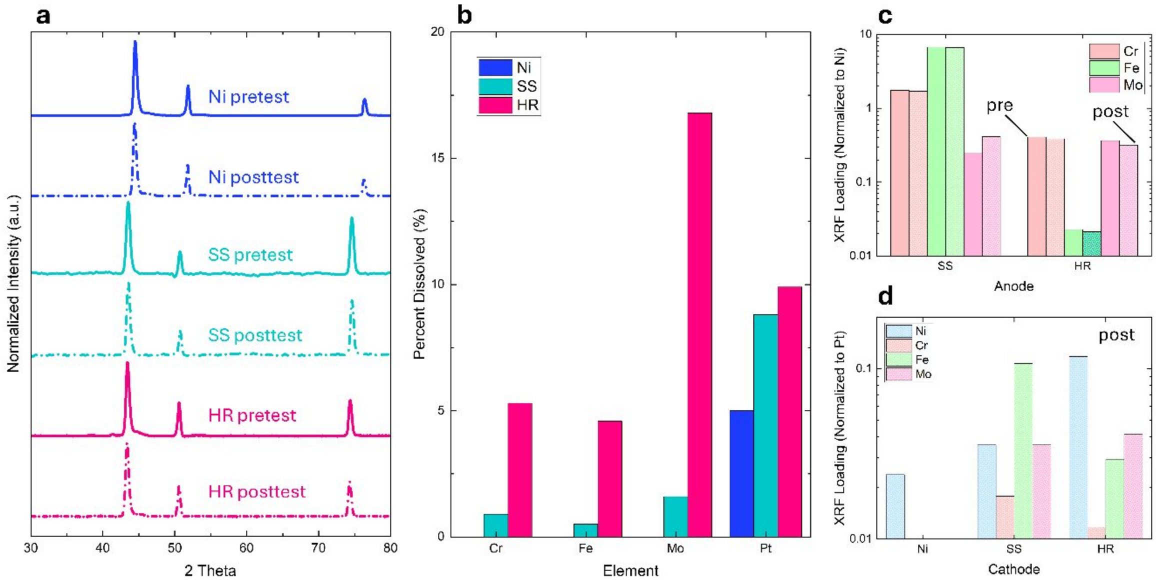

Figure S24. Characterization of the Ni 250, SS, and HR PTLs after the 100-h tests in **Figure 8**. (**a**) Comparison of pre- (solid line) and posttest (dashed line) XRD for Ni 205 (blue), SS (teal), and HR (pink). (**b**) Mass dissolved in electrolyte as percentage of initial PTL mass/catalyst loading for Cr, Fe, Mo, and Pt calculated from ex situ ICP-MS measurements of the electrolyte. (**c**) Metal loadings from XRF for the anodes pre- (filled bar) and posttest (patterned bar) for Cr (light red), Fe (light green), and Mo (light pink) normalized to the Ni mass. (**d**) Posttest metal loadings at the cathode for Ni (light blue), Cr (light red), Fe (light green), and Mo (light pink) normalized to the Pt loading.
